# Extensive genomic diversity and selective conservation of virulence-determinants in enterohemorrhagic *Escherichia coli *strains of O157 and non-O157 serotypes

**DOI:** 10.1186/gb-2007-8-7-r138

**Published:** 2007-07-10

**Authors:** Yoshitoshi Ogura, Tadasuke Ooka, Asadulghani, Jun Terajima, Jean-Philippe Nougayrède, Ken Kurokawa, Kousuke Tashiro, Toru Tobe, Keisuke Nakayama, Satoru Kuhara, Eric Oswald, Haruo Watanabe, Tetsuya Hayashi

**Affiliations:** 1 Division of Bioenvironmental Science, Frontier Science Research Center, University of Miyazaki,5200 Kihara, Kiyotake, Miyazaki, 889-1692, Japan; 2 Division of Microbiology, Department of Infectious Diseases, Faculty of Medicine, University of Miyazaki,5200 Kihara, Kiyotake, Miyazaki, 889-1692, Japan; 3 Department of Bacteriology, National Institute for Infectious Diseases, 1-23-1 Toyama, Shinjuku, Tokyo, 162-8640, Japan; 4 UMR1225, INRA-ENVT, 23 chemin des Capelles, 31076 Toulouse, France; 5 Laboratory of Comparative Genomics, Graduate School of Information Science, Nara Institute of Science and Technology, 8916-5 Takayama, Ikoma, Nara, 630-0192, Japan; 6 Laboratory of Molecular Gene Technics, Department of Genetic Resources Technology, Faculty of Agriculture, Kyushu University, 6-10-1 Hakosaki, Fukuoka, 812-8581, Japan; 7 Division of Applied Bacteriology, Graduate School of Medicine, Osaka University, 2-2 Yamadaoka, Suita, Osaka, 565-0871, Japan

## Abstract

**Background:**

Enterohemorrhagic *Escherichia coli *(EHEC) O157 causes severe food-borne illness in humans. The chromosome of O157 consists of 4.1 Mb backbone sequences shared by benign *E. coli *K-12, and 1.4 Mb O157-specific sequences encoding many virulence determinants, such as Shiga toxin genes (*stx *genes) and the locus of enterocyte effacement (LEE). Non-O157 EHECs belonging to distinct clonal lineages from O157 also cause similar illness in humans. According to the 'parallel' evolution model, they have independently acquired the major virulence determinants, the *stx *genes and LEE. However, the genomic differences between O157 and non-O157 EHECs have not yet been systematically analyzed.

**Results:**

Using microarray and whole genome PCR scanning analyses, we performed a whole genome comparison of 20 EHEC strains of O26, O111, and O103 serotypes with O157. In non-O157 EHEC strains, although genome sizes were similar with or rather larger than O157 and the backbone regions were well conserved, O157-specific regions were very poorly conserved. Around only 20% of the O157-specific genes were fully conserved in each non-O157 serotype. However, the non-O157 EHECs contained a significant number of virulence genes that are found on prophages and plasmids in O157, and also multiple prophages similar to, but significantly divergent from, those in O157.

**Conclusion:**

Although O157 and non-O157 EHECs have independently acquired a huge amount of serotype- or strain-specific genes by lateral gene transfer, they share an unexpectedly large number of virulence genes. Independent infections of similar but distinct bacteriophages carrying these virulence determinants are deeply involved in the evolution of O157 and non-O157 EHECs.

## Abstract 2

Comparing the genomes of O157 and non-O157 enterohemorrhagic *Escherichia coli *(EHEC) strains reveals the selective conservation of a large number of virulence determinants.

## Background

*Escherichia coli *is a commensal intestinal inhabitant of vertebrates and rarely cause diseases except in compromised hosts. Several types of strains, however, cause diverse intestinal and extra-intestinal diseases in healthy humans and animals by means of individually acquired virulence factors [1]. Enterohemorragic *E. coli *(EHEC) is one of the most devastating pathogenic *E. coli*, which can cause diarrhea and hemorrhagic colitis with life-threatening complications, such as hemolytic uremic syndrome (HUS) [2]. Shiga toxin (Stx) is the key virulence factor responsible for the induction of hemorrhagic colitis with such complications [3]. In addition, typical EHEC strains possess a pathogenicity island called 'the locus of enterocyte effacement (LEE)', which encodes a set of proteins constituting type III secretion system (T3SS) machinery. The LEE also encodes several effector proteins secreted by the T3SS, and an adhesin called intimin (encoded by the *eaeA *gene). The system confers on the bacteria the ability to induce attaching and effacing (A/E) lesions on the host colonic epithelial cells, enabling it to colonize tightly at the lesions [4]. The LEE has also been found in enteropathogenic *E. coli *(EPEC), which cause severe diarrhea in infants, and in several other animal pathogens, including *Citrobacter rodentium *and rabbit EPEC [5,6]. It is also known that EHEC strains harbor a large plasmid encoding several virulence factors, such as enterohemolysin [2].

Our previous genome sequence comparison of O157:H7 strain RIMD 0509952 (referred to as O157 Sakai) with the benign laboratory strain K-12 MG1655 revealed that the O157 Sakai chromosome is composed of 4.1 Mb sequences conserved in K-12, and 1.4 Mb sequences absent from K-12 (referred to as the backbone and S-loops, respectively) [7,8]. Importantly, most of the large S-loops are prophages and prophage-like elements, and O157 Sakai contains 18 prophages (Sp1-Sp18) and 6 prophage-like elements (SpLE1-SpLE6; these elements contain phage integrase-like genes but no other phage-related genes). These Sps and SpLEs carry most of the virulence-related genes of O157, including the *stx *genes (*stx1AB *on Sp15 and *stx2AB *on Sp5). The LEE pathogenicity island corresponds to SpLE4. Of particular importance is that, in addition to 7 LEE-encoded effectors, 32 proteins encoded in non-LEE loci have been identified as effectors secreted by LEE-encoded T3SS (non-LEE effectors) [9-15]. Among these, TccP has already been shown to play a pivotal role for the induction of A/E lesions in EHEC [16,17]. Others are also suspected to be involved in EHEC pathogenesis. Nearly all of these non-LEE effectors are encoded on the Sps and SpLEs [15].

We have recently performed a whole genome comparison of eight O157 strains by whole genome PCR scanning (WGPScanning) and comparative genomic hybridization (CGH) using O157 oligoDNA microarray analysis [18,19]. These analyses revealed that O157 strains are significantly divergent in the genomic structure and gene repertoire. In particular, Sp and SpLE regions exhibit remarkable diversity. We identified about 400 genes that are variably present in the O157 strains. They include several virulence-related genes, suggesting that some level of strain-to-strain variations in the potential virulence exist among O157 strains.

Although numerous EHEC outbreaks have been attributed to strains of the O157 serotype (O157 EHEC), it has increasingly been more frequently recognized that EHEC strains belonging to a wide range of other serotypes also cause similar gastrointestinal diseases in humans. Among these non-O157 EHECs, O26, O111, and O103 are the serotypes most frequently associated with human illness in many countries [20]. By multilocus sequencing typing (MLST) of housekeeping genes, Reid *et al*. [21] have shown that these non-O157 EHEC strains belong to clonal groups distinct from O157 EHEC. Based on this finding, they proposed a 'parallel' evolution model of EHEC; each EHEC lineage has independently acquired the same major virulence factors, *stx*, LEE, and plasmid-encoded enterohemolysin [21]. However, our knowledge on the prevalence of virulence factors among non-O157 EHEC strains is very limited. Many other virulence factors found on the O157 genome, such as fimbrial and non-fimbrial adhesins, iron uptake systems, and non-LEE effectors, are also thought to be required for the full virulence of EHEC, but their prevalence among non-O157 EHEC strains has not yet been systematically analyzed. Differences (or conservation) in the genomic structure between O157 and non-O157 EHEC strains are also yet to be determined.

In this study, we selected 20 non-O157 EHEC strains, 8 of which belong to O26, six to O111, and six to O103 serotypes, and performed a whole genome comparison with O157 EHEC strains by O157 oligoDNA microarray and WGPScanning. Our data indicate that the backbone regions are highly conserved also in non-O157 EHEC strains, while most S-loops are very poorly conserved. Among the genes on S-loops, only 8.5% were detected in all the EHEC strains examined, and around 20% were fully conserved in each non-O157 serotype. Besides, we found that the genome sizes of non-O157 EHEC strains are similar or rather larger than those of O157 strains, indicating that non-O157 EHEC strains have a huge amount of serotype- or strain-specific genes. Interestingly, virulence-related genes, particularly those for non-LEE effectors and non-fimbrial adhesions, were relatively well conserved in the non-O157 EHEC strains.

## Results

### Phylogeny and other features of non-O157 EHEC strains

EHEC strains used in this study were isolated from patients in Japan, Italy, or France (Table 1). The *Xba*I digestion patterns examined by pulsed field gel electrophoresis (PFGE) showed that the genomic DNA of EHEC strains is significantly divergent (Additional data file 1), while all possess *stx1 *and/or *stx2 *genes, and the *eaeA *gene encoding intimin (see 'Detection and subtyping of *stx *and *eaeA *genes' in Materials and methods). The results of the fluorescent actin staining (FAS) assay [22] indicated that all strains are potentially capable of inducing A/E lesions except for O111 strain 1. The efficiency, however, somewhat varied from strain-to-strain (data not shown).

**Table 1:** EHEC strains tested in this study

No.	Strain	Serotype	Source	Country	Symptoms	Shiga toxin	Intimin type
Sakai	RIMD 0509952	O157:H7	Human	Japan	(Sequenced strain)	*stx1*, *stx2*	γ1
O157 #2	980938	O157:H7	Human	Japan	Abdominal pain, fever	*stx1*, *stx2vh-b*	γ1
O157 #3	980706	O157:H7	Human	Japan	Diarrhea, bloody stool, abdominal pain	*stx1*, *stx2*, *stx2vh-a*	γ1
O157 #4	990281	O157:H7	Human	Japan	Asymptomatic carrier	*stx2vh-a*	γ1
O157 #5	980551	O157:H7	Human	Japan	Diarrhea, bloody stool	*stx1*, *stx2*	γ1
O157 #6	990570	O157:H7	Human	Japan	Diarrhea, bloody stool, fever	*stx2vh-a*	γ1
O157 #7	981456	O157:H7	Human	Japan	Diarrhea	*stx1*, *stx2vh-a*	γ1
O157 #8	982243	O157:H-	Human	Japan	Diarrhea, fever	*stx1*, *stx2vh-a*	γ1
O157 #9	981795	O157:H7	Human	Japan	Diarrhea, bloody stool, abdominal pain	*stx1*, *stx2*	γ1
O26 #1	11044	O26:H11	Human	Japan	Diarrhea, bloody stool	*stx1*	β1
O26 #2	11368	O26:H11	Human	Japan	Diarrhea	*stx1*	β1
O26 #3	11656	O26:H-	Human	Japan	Diarrhea, fever	*stx1*	β1
O26 #4	12719	O26:H-	Human	Japan	Diarrhea	*stx1*	β1
O26 #5	12929	O26:H-	Human	Japan	Diarrhea	*stx1*	β1
O26 #6	13065	O26:H11	Human	Japan	Diarrhea, abdominal pain	*stx1*	β1
O26 #7	13247	O26:H11	Human	Japan	Diarrhea, abdominal pain	*stx1*	β1
O26 #8	ED411	O26:H11	Human	Italy		*stx2*	β1
O111 #1	11109	O111:H-	Human	Japan	Diarrhea, abdominal pain	*stx1*	γy
O111 #2	11128	O111:H-	Human	Japan	Diarrhea, bloody stool	*stx1*, *stx2*	γy
O111 #3	11619	O111:H-	Human	Japan	Asymptomatic carrier	*stx1*, *stx2*	γy
O111 #4	11788	O111:H-	Human	Japan	Diarrhea	*stx1*	γy
O111 #5	13369	O111:H-	Human	Japan	Diarrhea, abdominal pain, bloody stool	*stx1*	γy
O111 #6	ED71	O111:H-	Human	Italy		*stx1*	γy
O103 #1	10828	O103:H2	Human	Japan	Diarrhea, abdominal pain	*stx1*	ε
O103 #2	11117	O103:H2	Human	Japan	Diarrhea, fever	*stx1*	ε
O103 #3	11711	O103:H2	Human	Japan	Diarrhea, fever	*stx1*	ε
O103 #4	11845	O103:H2	Human	Japan	Diarrhea, abdominal pain	*stx1*	ε
O103 #5	12009	O103:H2	Human	Japan	Diarrhea, bloody stool	*stx1*, *stx2*	ε
O103 #6	PMK5	O103:H2	Human	France	HUS	*stx1*	ε

The MLST analysis using seven housekeeping genes (*aspC*, *clpX*, *fadD*, *icdA*, *lysP*, *mdh*, and *uidA*) indicated that strains belonging to the O157, O26, O111, and O103 serotypes were clustered into three different phylogenic groups (O26 and O111 strains were clustered together; Additional data file 2). This result is basically consistent with those from previous MLST analyses using different genetic loci [21,23]. The type of intimin was classified as γ1, β1, γ2, and ε for O157, O26, O111, and O103, respectively.

### Chromosome sizes and plasmid profiles

The I-*Ceu*I digestion of chromosomal DNA yielded seven fragments in 26 out of 29 EHEC strains (data not shown). Because I-*Ceu*I specifically cleaves a 19 base-pair sequence in the 23S ribosomal RNA gene, it demonstrated that these strains have seven copies of the ribosomal operon (*rrn*), as in K-12 and O157. Estimated chromosome sizes of these strains were all much larger than that of K-12, with diverged sizes ranging from 5,102 to 5,945 kb (Table 2). O111 and O103 strains contained slightly smaller chromosomes than O157 strains. In contrast, most O26 strains contained relatively larger chromosomes. We could not estimate the chromosome sizes in two O157 strains (2 and 9) and one O103 strain (4), because all or the largest fragments repeatedly exhibited smear patterns.

**Table 2:** Estimated genome sizes of EHEC strains

					Estimated sizes (kb)																											
					
	K-12*		Sakai*		O157								O26								O111						O103					
						
	*In silico*	Exp	*In silico*	Exp	#2	#3	#4	#5	#6	#7	#8	#9	#1	#2	#3	#4	#5	#6	#7	#8	#1	#2	#3	#4	#5	#6	#1	#2	#3	#4	#5	#6
I-*ceu*I-fragmant no.																																
1	2,498	2,686	3,216	3,191	ND	3,342	3,325	3,277	3,226	3,358	3,325	ND	3,185	3,386	3,345	3,414	3,571	3,513	3,630	3,374	2,941	3,044	2,912	2,898	2,884	2,814	2,911	2,959	3,291	ND	2,923	2,961
2	698	687	712	720	722	722	713	713	693	718	708	ND	777	777	782	823	751	787	782	734	824	803	808	808	803	808	889	923	941	872	883	761
3	657	649	709	707	698	679	679	657	670	679	674	ND	746	751	751	741	720	720	720	720	698	698	698	693	693	698	709	720	797	714	756	712
4	521	525	579	591	574	574	574	574	574	582	574	ND	382	382	458	382	385	385	385	537	519	519	519	519	519	519	517	517	346	521	362	514
5	131	127	144	142	144	142	179	142	142	144	144	ND	295	295	301	295	298	298	298	143	140	137	137	135	135	135	137	136	317	133	320	136
6	94	83	96	89	89	88	88	88	91	88	89	ND	97	97	96	97	97	97	97	99	92	92	92	91	86	88	98	101	97	98	97	93
7	41	41	41	41	43	42	42	42	42	42	42	ND	41	41	41	41	41	41	33	41	41	41	41	41	41	41	41	43	43	43	43	43
Chromosome total	4,640	4,797	5,498	5,480	ND	5,589	5,600	5,492	5,437	5,610	5,556	ND	5,524	5,731	5,773	5,794	5,864	5,842	5,945	5,647	5,256	5,334	5,207	5,185	5,160	5,102	5,303	5,398	5,833	ND	5,384	5,220
																																
Plasmid no.																																
1			93	93	93	93	101	93	93	93	93	ND	7	85	91	98	98	98	98	137	77	205	125	81	87	155	74	ND	89	89	72	52
2			3	3					6	7	3	ND		63	65	73	49		91	107		98	77	51	47	7		ND	72	63		
3									3			ND		6		4	7		68	25		78	7	7	7	5		ND				
4												ND		4					7	3		8		5	5			ND				
5												ND										7						ND				
Plasmid total	-	-	96	96	93	93	101	93	102	99	95	ND	7	158	156	175	154	98	263	273	77	395	208	144	145	166	74	ND	160	152	72	52
																																
Genome total	4,640	4,797	5,594	5,576	NE	5,682	5,701	5,585	5,539	5,709	5,651	ND	5,530	5,889	5,929	5,969	6,018	5,940	6,208	5,920	5,333	5,729	5,415	5,328	5,305	5,268	5,377	ND	5,993	ND	5,456	5,273

*Lengths of each band estimated from experimental data and *in silico *analyses are shown. ND, not detected.

Plasmid profiles indicated that all but one O157 strain contain one large plasmid of a similar size (Table 2; Additional data file 3). All of the non-O157 EHEC strains also contained at least one large plasmid except for O26 strain 1 (one small plasmid was present) and O103 strain 2 (no plasmid was detected). Several O26 and O111 strains possessed two or three large plasmids. The estimated total genome sizes of EHEC strains ranged from 5.27 Mb to 6.21 Mb.

### Overview of the CGH analysis of non-O157 EHEC

**Figure 1:**
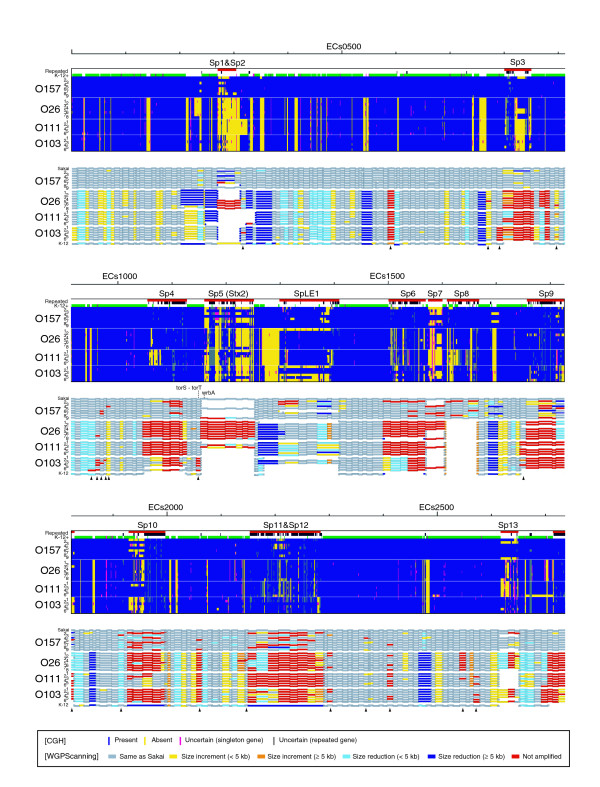
Summary of the CGH and WGPScanning analyses of O157 and non-O157 EHEC strains. Results from the CGH analysis of 29 EHEC strains using an O157 oligoDNA microarray are shown in the upper half of each segment, and those from the genome structural analysis by the WGPScaning method in the lower half. Above the CGH data, genes on prophages (Sps), prophage-like elements (SpLEs), and plasmids are indicated in red (the first row), repeated genes in black (the second row), and genes conserved or partially conserved in K-12 in green or pink, respectively (the third row). Genes judged as present in the CGH analysis are indicated in blue and those absent in yellow. Singleton and repeated genes classified as 'uncertain' are indicated in pink and gray, respectively. Results from the WGPScanning analysis are presented as follows. Segments of the same sizes as those from O157 Sakai are indicated in gray, and those with large (≥5 kb) and small (<5 kb) size reductions in blue and light blue, respectively. The segments with large (≥5 kb) and small (<5 kb) size increments are indicated in orange and yellow, respectively, and those not amplified in red. When Sps, SpLEs, or their corresponding elements were not integrated in relevant loci, such regions are depicted as blank areas. The segments containing potential integration sites for large genomic elements are indicated by arrowheads. Positions of known and newly identified integration sites for Stx phages and LEE elements are indicated between the panels for the CGH and WGPScanning data. In this figure, each segment is not drawn to scale but to the gene position in the data presentation of the CGH analyses. The data from the first half of EHEC chromosomes are shown in this figure, and those from the second half and plasmids in Figure 2.

**Figure 2:**
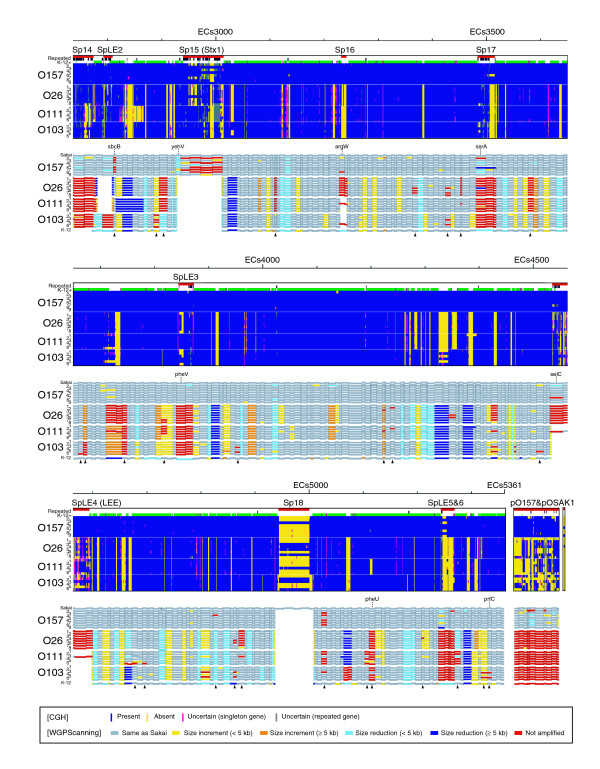
Summary of the CGH and WGPScanning analyses of O157 and non-O157 EHEC strains. The data from CGH and WGPScanning analyses of 29 EHEC strains are shown. The data from the second half of EHEC chromosomes and plasmids are shown in this figure. See the legend of Figure 1 for details.

We analyzed the gene contents of non-O157 EHEC strains by using the O157 oligoDNA microarray, and compared the results with those of O157 strains in our previous report [18] (Figures 1 and 2). More Sakai genes were absent from the non-O157 EHEC strains. In O157 strains, the absent genes were found mostly in Sp and SpLE regions, but in non-O157 EHEC strains, they were found not only in Sp and SpLE regions but also in various S-loops. The conservation tended to exhibit a serotype-specific pattern, but remarkable strain-to-strain diversity was also observed in each serotype.

To more precisely analyze the CGH data, we categorized the Sakai genes into three groups [18]. Since most Sakai genes were represented by two oligonucleotide probes in our microarray, we first classified the probes into two groups by their homologies to the K-12 genome sequence; those with ≥90% identity into 'conserved in K-12' probes and others into 'Sakai-specific' probes. Each gene was then classified into 'conserved in K-12' genes, 'partly conserved in K-12' genes (genes represented by one 'conserved in K-12' probe and one 'Sakai-specific' probe), or 'Sakai-specific' genes. Repeated gene families that occurred in O157 Sakai more than once were analyzed separately from singleton genes (see Materials and methods for details on the classification and the presence or absence determination).

**Figure 3:**
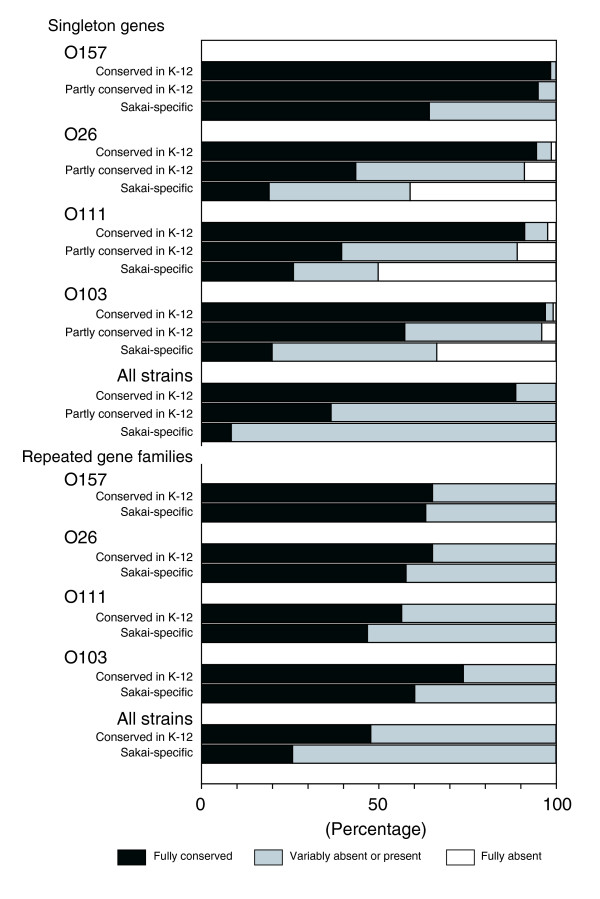
Conservation of O157 Sakai genes in O157 and non-O157 EHEC strains. The data from CGH analyses of O157 and non-O157 EHEC strains using an O157 Sakai oligoDNA microarray are summarized. Among the 4,905 singleton genes on the O157 Sakai genome, 3,651 were categorized as 'conserved in K-12', 101 as 'partly conserved in K-12', and 1,153 as 'Sakai-specific'. Among the 151 repeated gene families, 23 were categorized as 'conserved in K-12' and 128 as 'Sakai-specific'. Genes that were judged as 'present' in all the tested strains were categorized as 'Fully conserved' genes, those judged as 'absent' in all the strains as 'Fully absent' genes, and others as 'Variably absent or present' genes. In the CGH analysis, because repeated gene families with reduced copy numbers were often judged as 'absent', all the repeated gene families judged as 'absent' were categorized as 'uncertain'. See Additional data file 4 for further details.

'Conserved in K-12' singleton genes were highly conserved in all serotypes: 3,596 (98.5%), 3,450 (94.5%), 3,331 (91.2%), and 3,542 (97.0%) out of 3,651 genes were fully conserved in O157, O26, O111 and O103, respectively, and 3,240 (88.7%) in all the test strains (Figure 3; Additional data file 4). 'Sakai-specific' singleton genes were relatively well conserved in O157 strains, but very poorly in non-O157 EHEC strains: 741 (64.3%), 221 (19.2%), 300 (26.0%), and 231 (20.0%) out of 1,153 genes were fully conserved in O157, O26, O111, and O103, respectively. Only 98 (8.5%) were conserved in all the test strains.

Among the 4,905 singleton genes, 101 were categorized as 'partly conserved in K-12' genes. They included 81 genes that are encoded on the backbone and 20 genes on S-loops or backbone/S-loop junctions. In O157, all but 5 (95.0%) of the 'partly conserved in K-12' genes were fully conserved. In non-O157 EHECs, however, many 'partly conserved in K-12' genes were categorized as 'uncertain' (7 to 42 genes in each non-O157 EHEC strain, 28 genes on average), because only one of the two probes yielded positive results. Therefore, only 44 (43.6%), 40 (39.6%), and 58 (57.4%) were fully conserved in O26, O111, and O103, respectively (Figure 3; Additional data file 4). This result suggests that most of the 'partly conserved in K-12' genes are present in the non-O157 EHEC strains but many have significantly divergent sequences from those of O157 Sakai.

O157 Sakai contains many repeated genes (542 out of 5,447 genes), such as transposase- and phage-related genes. They can be grouped into 151 families. Compared with the singleton genes, the repeated gene families were relatively well conserved in non-O157 EHECs. About half of the 'conserved in K-12' repeated gene families (11 out of the 23 families (47.8%)) were fully conserved in all the test strains, and 81 (63.3%), 74 (57.8%), 60 (46.9%), and 77 (60.2%) out of the 128 'Sakai-specific' repeated gene families were fully conserved in O157, O26, O111, and O103, respectively (Figure 3; Additional data file 4). Because most of the repeated genes were from lambda-like prophages and IS elements [8,18], this result indicates that non-O157 EHEC strains also contain multiple lambda-like prophages and IS elements very similar to those found in O157 Sakai.

### Absent 'conserved in K-12' genes in EHEC strains

Among the 3,651 'conserved in K-12' singleton genes, 224 (6.1%) were absent in at least one test strain. These genes were found to be absent more frequently in non-O157 EHEC strains than in O157 strains: 75 genes (2.1%) in O26 strains, 184 (5.0%) in O111, and 61 (1.7%) in O103, while only 37 (1.0%) in O157 (here we counted only the genes that were judged as 'absent' in at least one strain; therefore, these results do not include the genes that were 'uncertain' in some strains but 'absent' in no strain). These genes were dispersed on the chromosome and belonged to various functional categories (Additional data file 5); but as expected, none of them was listed as essential, either in the 'profiling of *E. coli *chromosome' (PEC) database [24] or in a systematic single-gene deletion study of *E. coli *K-12 [25]. We also identified 46, 83, and 30 'conserved in K-12' singleton genes that are fully absent in O26, O111, and O103, respectively. Among these, 22 genes, which are located in 12 different chromosomal loci, were absent in all non-O157 EHEC strains, and 10, 44, and 3 genes were specifically missing in O26, O111, and O103, respectively.

### Conservation of 'Sakai-specific' genes in non-O157 EHEC strains

**Figure 4:**
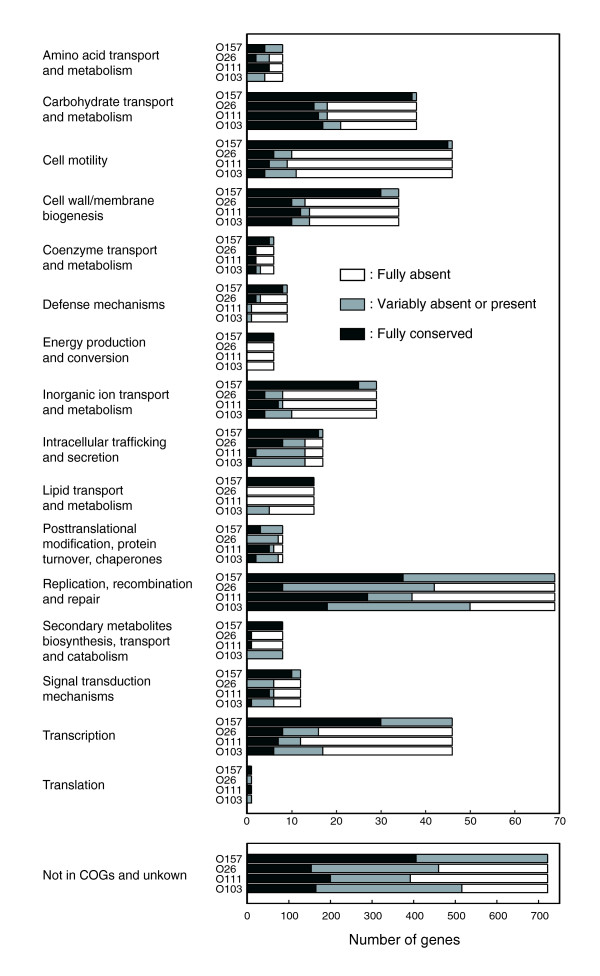
Conservation of 'Sakai-specific' singleton genes in each functional group. 'Sakai-specific' singleton genes were categorized according to the COG classification. In each functional category, the numbers of genes fully conserved, variably absent or present, and fully absent are shown for each serotype.

We categorized 'Sakai-specific' singleton genes according to the COG (clusters of orthologous groups of proteins) classification [26], and analyzed the gene conservation of each functional category (Figure 4). In O157, most genes were well conserved in all categories. Many genes for 'replication, recombination and repair' and for 'transcription' were variably present among O157 strains, but most of them were on Sps and SpLEs. In the non-O157 serotypes, however, the 'Sakai-specific' singleton genes belonging to almost every COG functional category exhibited poor conservation (many were classified as 'Fully absent'). The level of conservation was similar to that observed for the four sequenced pathogenic *E. coli *strains of different pathotypes [27-30] (Additional data file 4).

A relatively large number of genes for 'carbohydrate transport and metabolism' were fully conserved in non-O157 EHECs. Among these, genes for the sugar ABC transporter system (ECs0374-0378), and the N-acetylgalactosamine-specific PTS system (ECs4013-4014), and two genes for sugar utilization (ECs3242: fructokinase and ECs3243: sucrose-6 phosphate hydrolase) were conserved in all the tested strains. A relatively large number of genes for the 'cell wall/membrane biogenesis' category were also fully conserved. Most of them were the genes for lipopolysaccharide core biosynthesis (ECs2831 and ECs2836-2845). This is consistent with the fact that four serotypes examined here share the same core type (R3) [31,32].

SpLE1 carries gene clusters for urease (ECs1321-1327) and tellurite resistance (ECs1343, 1351-1358). In an earlier report, the urease genes were found specifically associated with EHEC strains irrespective of their serotypes [33]. Our present data, however, demonstrate that five EHEC strains (one O157, one O26, and three O103 strains) lack the urease genes. The tellurite resistance genes were also well conserved in non-O157 EHECs but absent in one O26 and two O103 strains.

### Distribution of O157 Sakai virulence-related genes in non-O157 EHECs

In the COG classification, many of the virulence-related genes were classified into the 'not in COGs' category. We thus picked up all the known or suspected O157 virulence-related genes, and analyzed their conservation in non-O157 EHECs. Fimbria are important for virulence as an initial attachment factors to the host intestine. The O157 Sakai genome contained 14 fimbrial biosynthesis gene clusters (loci 1 to 14), all of which were completely conserved in every O157 strain except for strain 8, in which locus 11 was partially conserved (Table 3). Among the 14 clusters, four (loci 3, 5, 7, and 14) were completely conserved in K-12 and three (loci 1, 8, and 11) partially conserved. These seven loci were also completely or partially conserved in the non-O157 EHEC strains, suggesting that these gene clusters are widely conserved in various *E. coli *strains irrespective of their pathotypes. Genes on the remaining seven loci were almost completely absent in all non-O157 serotypes. Only loci 9 and 10 were partially conserved in several non-O157 EHEC strains. Thus, we may regard them as O157-specifc fimbrial gene clusters.

**Table 3:** Conservation of fimbrial loci in each EHEC strain

		K-12	O157								O26								O111						O103					
						
Locus no.	ECs number		#2	#3	#4	#5	#6	#7	#8	#9	#1	#2	#3	#4	#5	#6	#7	#8	#1	#2	#3	#4	#5	#6	#1	#2	#3	#4	#5	#6
1	ECs0019-0024	p	+	+	+	+	+	+	+	+	p	p	p	p	p	p	p	p	p	p	p	p	p	p	p	p	p	p	p	p
2	ECs0139-0145	-	+	+	+	+	+	+	+	+	-	-	-	-	-	-	-	-	-	-	-	-	-	-	-	-	-	-	-	-
3	ECs0592-0597	+	+	+	+	+	+	+	+	+	+	+	+	+	+	+	+	+	+	+	+	+	+	+	+	+	+	+	+	+
4	ECs0741-0744	-	+	+	+	+	+	+	+	+	-	-	-	-	-	-	-	-	-	-	-	-	-	-	-	-	-	-	-	-
5	ECs1021-1028	+	+	+	+	+	+	+	+	+	+	+	+	+	+	+	+	+	+	+	+	+	+	+	+	+	+	+	+	+
6	ECs1276-1281	-	+	+	+	+	+	+	+	+	-	-	-	-	-	-	-	-	-	-	-	-	-	-	-	-	-	-	-	-
7	ECs0267&1414-1421	+	+	+	+	+	+	+	+	+	+	+	+	+	+	+	+	+	+	+	+	+	+	+	+	+	+	+	+	+
8	ECs2107-2114	p	+	+	+	+	+	+	+	+	+	+	+	+	+	+	+	+	+	+	+	+	+	+	p	p	+	p	p	+
9	ECs2914-2918	-	+	+	+	+	+	+	+	+	-	-	-	-	-	-	-	-	-	-	-	-	-	-	-	-	p	-	-	p
10	ECs3216-3222	-	+	+	+	+	+	+	+	+	p	-	-	p	-	-	p	p	p	p	-	p	-	p	p	p	p	p	p	p
11	ECs4020-4023&4026	p	+	+	+	+	+	+	p	+	+	+	+	+	+	+	+	+	+	+	+	+	+	+	+	+	+	+	+	+
12	ECs4426-4431	-	+	+	+	+	+	+	+	+	-	-	-	-	-	-	-	-	-	-	-	-	-	-	-	-	-	-	-	-
13	ECs4665-4670	-	+	+	+	+	+	+	+	+	-	-	-	-	-	-	-	-	-	-	-	-	-	-	-	-	-	-	-	-
14	ECs5271-5279	+	+	+	+	+	+	+	+	+	+	+	+	+	+	+	+	+	+	p	+	+	+	+	+	+	+	+	+	+

Symbols: '+' indicates a locus where all genes were conserved; '-' a locus where all genes were absent; and 'P' a locus where one or more genes, but not all genes, were absent. Genes judged as 'uncertain' were not considered.

In addition to the fimbrial genes, 14 Sakai genes have been demonstrated or suspected to encode non-fimbrial adhesins (Table 4). They were relatively well conserved in the non-O157 EHEC strains. 'Regulators' and 'Toxins and their activators' showed similar levels of conservation as the genes related to adhesion (Table 4).

**Table 4:** Conservation of Sakai virulence-related genes in EHECs and other sequenced pathogenic *E. coli *strains

				In silico*				No. of strains conserved^†^			
					
Gene	Location	Conservation in K-12	Common name/description	CFT073	UTI89	536	APEC	O157 (8)	O26 (8)	O111 (6)	O103 (6)
**Genes related to adhsion (not fimbrial genes)**											
ECs0336	S-loop20	Partly conserved	Putative invasin	**+**	**+**	**+**	**+**	**[8]**	**[8]**	**[6]**	**[6]**
ECs0350	S-loop23	Absent	HmwA-like protein	**-**	**-**	**+**	**-**	**(7)**	0	0	0
ECs0362	S-loop24	Absent	AidA-I adhesin-like protein	**-**	**-**	**-**	**-**	**[8]**	**[8]**	**[6]**	**[6]**
ECs0548	S-loop43	Absent	Saa-like protein	**-**	**+**	**-**	**+**	**[8]**	0	0	0
ECs1360	SpLE1	Absent	Iha adhesin	**+**	**-**	**-**	**-**	**[8]**	**[8]**	**[6]**	2
ECs1396	SpLE1	Conserved	AidA-I adhesin-like protein	**-**	**-**	**-**	**-**	**(7)**	**[8]**	0	**[6]**
ECs1772	Sp9	Absent	Paa	**-**	**-**	**-**	**-**	**[8]**	**(7)**	**[6]**	4
ECs2006	Backbone	Absent	BigA-like protein	**-**	**-**	**-**	**-**	**[8]**	0	0	0
ECs2007	Backbone	Absent	BigB-like protein	**-**	**-**	**-**	**-**	**[8]**	0	0	0
ECs2567	Backbone	Conserved	Putative adhesin	**+**	**+**	**+**	**+**	**[8]**	**[8]**	**[6]**	**[6]**
ECs3860	SpLE3	Absent	Efa1 (interrupted)	**-**	**-**	**-**	**-**	**(7)**	**[8]**	**[6]**	**[6]**
ECs3861	SpLE3	Absent	Efa1 (partial)	**-**	**-**	**-**	**-**	**(7)**	**[8]**	**[6]**	**[6]**
ECs4559	SpLE4 (LEE)	Absent	Gamma intimin	**-**	**-**	**-**	**-**	**[8]**	0	0	0
ECs5290	S-loop288	Absent	Putative invasin	**-**	**-**	**-**	**-**	**(7)**	0	0	0
											
**Genes confer resistance to host immune response**											
ECs0218	S-loop14	Absent	IcmF-like protein	**-**	**-**	**-**	**-**	**[8]**	**[8]**	**[6]**	**[6]**
ECs1236	Sp5	Absent	Lom	**-**	**-**	**-**	**-**	5	1	2	0
ECs1312	SpLE1	Absent	TraT	**-**	**-**	**-**	**-**	**(7)**	**(7)**	0	3
ECs1956	Sp10	Absent	IrsA-like protein	**-**	**-**	**-**	**+**	5	1	**(5)**	1
ECs3850	SpLE3	Absent	PagC-like protein	**-**	**-**	**-**	**-**	**[8]**	0	**[6]**	2
RF001	Sp5, 8	Conserved	Bor	**+**	**+**	**+**	**+**	6	**[8]**	1	5
RF098	Sp4, 10	Absent	Copper/zinc-superoxide dismutase	**-**	**-**	**-**	**-**	4	1	**(5)**	**[6]**
RF115	Sp3, 4, 8 - 12, 14, 15	Absent	Lom	**-**	**-**	**-**	**+**	**[8]**	**[8]**	**[6]**	**[6]**
											
**Toxins and activators**											
ECs0541	S-loop42	Absent	RTX-like protein	**-**	**-**	**-**	**-**	**[8]**	0	0	0
ECs0542	S-loop42	Absent	RTX-like protein	**-**	**-**	**-**	**-**	**[8]**	0	0	0
ECs0814	Sp3	Absent	SfpA (systemic factor protein A)-like protein	**-**	**-**	**-**	**-**	**[8]**	**[8]**	**[6]**	**[6]**
ECs1205	Sp5	Absent	Shiga toxin 2 subunit A	**-**	**-**	**-**	**-**	4	1	2	1
ECs1206	Sp5	Absent	Shiga toxin 2 subunit B	**-**	**-**	**-**	**-**	3	1	2	1
ECs1282	S-loop71	Absent	Hemagglutinin/hemolysin - related protein	**-**	**-**	**-**	**-**	**[8]**	0	0	0
ECs1283	S-loop71	Absent	Hemolysin activator - related protein	**-**	**-**	**-**	**-**	**[8]**	0	0	0
ECs1382	SpLE1	Absent	HecB-like protein	**-**	**-**	**-**	**-**	5	**(7)**	**[6]**	2
ECs1652	Sp8	Absent	Putative catalase	**-**	**-**	**-**	**-**	**[8]**	0	0	0
ECs1677	Backbone	Conserved	Hemolysin E	**-**	**-**	**-**	**-**	**[8]**	**[8]**	**[6]**	**[6]**
ECs2973	Sp15	Absent	Stx1B	**-**	**-**	**-**	**-**	6	**(7)**	**[6]**	**[6]**
ECs2974	Sp15	Absent	Stx1A	**-**	**-**	**-**	**-**	6	**(7)**	**[6]**	**[6]**
											
**Regulators**											
ECs1274	s-loop71	Absent	GrvA	**-**	**-**	**-**	**-**	**[8]**	0	0	0
ECs1388	SpLE1	Absent	PchD	**+**	**-**	**-**	**+**	4	**(7)**	2	2
ECs1588	Sp7	Absent	PchE	**-**	**-**	**-**	**-**	**(7)**	**[8]**	0	4
ECs3105	Backbone	Conserved	RcsD	**+**	**+**	**+**	**+**	**[8]**	**[8]**	**[6]**	**[6]**
ECs3106	Backbone	Conserved	RcsB	**+**	**+**	**+**	**+**	**[8]**	**[8]**	**[6]**	**[6]**
ECs3107	Backbone	Conserved	RcsC	**+**	**+**	**+**	**+**	**[8]**	**[8]**	**[6]**	**[6]**
ECs3720	ETT2	Absent	EtrA	**-**	**-**	**-**	**-**	**[8]**	**[8]**	4	4
ECs3734	ETT2	Absent	EivF	**-**	**-**	**-**	**-**	**[8]**	0	0	0
ECs4577	SpLE4 (LEE)	Absent	GrlA	**-**	**-**	**-**	**-**	**[8]**	**[8]**	4	**(5)**
ECs4578	SpLE4 (LEE)	Absent	GrlR	**-**	**-**	**-**	**-**	**[8]**	**[8]**	4	4
ECs4588	SpLE4 (LEE)	Absent	Ler	**-**	**-**	**-**	**-**	**[8]**	**[8]**	**(5)**	**[6]**
RF132	Sp4, 11, 14	Absent	PchA, B, C	**-**	**-**	**-**	**-**	**[8]**	**(7)**	**[6]**	**[6]**
											
**Secretion machineries**											
ECs0540	S-loop42	Absent	CyaE-like protein	**-**	**-**	**-**	**-**	**[8]**	0	0	0
ECs0543	S-loop43	Absent	Putative RTX toxin secretion ATP-binding protein	**-**	**-**	**-**	**-**	**[8]**	0	0	0
ECs0544	S-loop44	Absent	Putative RTX toxin secretion membrane fusion protein	**-**	**-**	**-**	**-**	**[8]**	0	0	0
ECs3716	ETT2	Absent	EprK	**-**	**-**	**-**	**-**	**[8]**	**[8]**	**(5)**	**(5)**
ECs3717	ETT2	Absent	EprJ	**-**	**-**	**-**	**-**	**[8]**	**[8]**	4	**(5)**
ECs3718	ETT2	Absent	EprI	**-**	**-**	**-**	**-**	**[8]**	**[8]**	**(5)**	4
ECs3719	ETT2	Absent	EprH	**-**	**-**	**-**	**-**	**[8]**	**[8]**	4	**(5)**
ECs3721	ETT2	Absent	EpaS	**-**	**-**	**-**	**-**	**[8]**	**[8]**	**(5)**	**(5)**
ECs3722	ETT2	Absent	EpaR2	**-**	**-**	**-**	**-**	**[8]**	**[8]**	4	4
ECs3723	ETT2	Absent	EpaR1	**-**	**-**	**-**	**-**	**[8]**	**[8]**	**(5)**	4
ECs3724	ETT2	Absent	EpaQ	**-**	**-**	**-**	**-**	**[8]**	**[8]**	**(5)**	**(5)**
ECs3725	ETT2	Absent	EpaP	**-**	**-**	**-**	**-**	**[8]**	**[8]**	4	4
ECs3726	ETT2	Absent	EpaO	**-**	**-**	**-**	**-**	**[8]**	1	0	2
ECs3727	ETT2	Absent	EivJ	**-**	**-**	**-**	**-**	**[8]**	0	0	0
ECs3729	ETT2	Absent	EivI	**-**	**-**	**-**	**-**	**[8]**	0	0	0
ECs3730	ETT2	Absent	EivC	**-**	**-**	**-**	**-**	**[8]**	0	0	0
ECs3731	ETT2	Absent	EivA	**-**	**-**	**-**	**-**	**[8]**	0	0	0
ECs3732	ETT2	Absent	EivE	**-**	**-**	**-**	**-**	**[8]**	0	0	0
ECs3733	ETT2	Absent	EivG	**-**	**-**	**-**	**-**	**[8]**	0	0	0
ECs4551	SpLE4 (LEE)	Absent	Orf29	**-**	**-**	**-**	**-**	**[8]**	3	**[6]**	**(5)**
ECs4552	SpLE4 (LEE)	Absent	EscF	**-**	**-**	**-**	**-**	**[8]**	**[8]**	**[6]**	**[6]**
ECs4553	SpLE4 (LEE)	Absent	CesD2	**-**	**-**	**-**	**-**	**[8]**	**[8]**	**[6]**	**[6]**
ECs4555	SpLE4 (LEE)	Absent	EspD	**-**	**-**	**-**	**-**	**[8]**	0	0	1
ECs4556	SpLE4 (LEE)	Absent	EspA	**-**	**-**	**-**	**-**	**[8]**	1	0	1
ECs4557	SpLE4 (LEE)	Absent	SepL	**-**	**-**	**-**	**-**	**[8]**	**[8]**	**[6]**	**(5)**
ECs4558	SpLE4 (LEE)	Absent	EscD	**-**	**-**	**-**	**-**	**[8]**	0	3	2
ECs4560	SpLE4 (LEE)	Absent	CesT	**-**	**-**	**-**	**-**	**[8]**	6	**[6]**	**[6]**
ECs4563	SpLE4 (LEE)	Absent	CesF	**-**	**-**	**-**	**-**	**[8]**	0	0	0
ECs4565	SpLE4 (LEE)	Absent	SepQ	**-**	**-**	**-**	**-**	**[8]**	0	0	1
ECs4566	SpLE4 (LEE)	Absent	Orf16	**-**	**-**	**-**	**-**	**[8]**	0	**(5)**	0
ECs4567	SpLE4 (LEE)	Absent	Orf15	**-**	**-**	**-**	**-**	**[8]**	6	**(5)**	4
ECs4568	SpLE4 (LEE)	Absent	EscN	**-**	**-**	**-**	**-**	**[8]**	4	**(5)**	3
ECs4569	SpLE4 (LEE)	Absent	EscV	**-**	**-**	**-**	**-**	**[8]**	6	**(5)**	2
ECs4570	SpLE4 (LEE)	Absent	Orf12	**-**	**-**	**-**	**-**	**[8]**	4	4	2
ECs4572	SpLE4 (LEE)	Absent	Rorf8	**-**	**-**	**-**	**-**	**[8]**	0	0	0
ECs4573	SpLE4 (LEE)	Absent	EscJ	**-**	**-**	**-**	**-**	**[8]**	3	4	2
ECs4574	SpLE4 (LEE)	Absent	SepD	**-**	**-**	**-**	**-**	**[8]**	0	4	2
ECs4575	SpLE4 (LEE)	Absent	EscC	**-**	**-**	**-**	**-**	**[8]**	**[8]**	**[6]**	4
ECs4576	SpLE4 (LEE)	Absent	CesD	**-**	**-**	**-**	**-**	**[8]**	5	4	3
ECs4579	SpLE4 (LEE)	Absent	Rorf3	**-**	**-**	**-**	**-**	**[8]**	1	4	2
ECs4580	SpLE4 (LEE)	Absent	EscU	**-**	**-**	**-**	**-**	**[8]**	**(7)**	**(5)**	4
ECs4581	SpLE4 (LEE)	Absent	EscT	**-**	**-**	**-**	**-**	**[8]**	**(7)**	4	3
ECs4582	SpLE4 (LEE)	Absent	EscS	**-**	**-**	**-**	**-**	**[8]**	**[8]**	**(5)**	**(5)**
ECs4583	SpLE4 (LEE)	Absent	EscR	**-**	**-**	**-**	**-**	**[8]**	**[8]**	4	**(5)**
ECs4584	SpLE4 (LEE)	Absent	Orf5	**-**	**-**	**-**	**-**	**[8]**	**[8]**	**[6]**	**(5)**
ECs4585	SpLE4 (LEE)	Absent	Orf4	**-**	**-**	**-**	**-**	**[8]**	**[8]**	4	**(5)**
ECs4586	SpLE4 (LEE)	Absent	Orf3	**-**	**-**	**-**	**-**	**[8]**	6	**[6]**	**(5)**
ECs4587	SpLE4 (LEE)	Absent	Orf2	**-**	**-**	**-**	**-**	**[8]**	3	1	2
											
**T3SS effectors (LEE encoded)**											
ECs4550	SpLE4 (LEE)	Absent	EspF1	**-**	**-**	**-**	**-**	**[8]**	0	0	0
ECs4554	SpLE4 (LEE)	Absent	EspB	**-**	**-**	**-**	**-**	**[8]**	0	0	0
ECs4561	SpLE4 (LEE)	Absent	Tir	**-**	**-**	**-**	**-**	**[8]**	0	0	0
ECs4562	SpLE4 (LEE)	Absent	Map	**-**	**-**	**-**	**-**	**[8]**	0	0	2
ECs4564	SpLE4 (LEE)	Absent	EspH	**-**	**-**	**-**	**-**	**[8]**	**[8]**	0	**[6]**
ECs4571	SpLE4 (LEE)	Absent	SepZ	**-**	**-**	**-**	**-**	**[8]**	0	0	0
ECs4590	SpLE4 (LEE)	Absent	EspG	**-**	**-**	**-**	**-**	**[8]**	1	3	2
											
**T3SS effectors (non-LEE encoded)**											
ECs0061	Backbone	Conserved	EspY1	**-**	**-**	**-**	**-**	**[8]**	0	0	0
ECs0847	Sp3	Absent	NleC	**-**	**-**	**-**	**-**	**[8]**	**(7)**	1	1
ECs0848	Sp3	Absent	NleH1-1	**-**	**-**	**-**	**-**	**[8]**	1	0	3
ECs0850	Sp3	Absent	NleD	**-**	**-**	**-**	**-**	**[8]**	0	0	4
ECs0876	S-loop57	Absent	EspX2	**-**	**-**	**-**	**-**	**[8]**	0	0	0
ECs1127	Sp4	Absent	EspK	**-**	**-**	**-**	**-**	**[8]**	**[8]**	**[6]**	**[6]**
ECs1560	Sp6	Absent	EspX7	**-**	**-**	**-**	**-**	**[8]**	**(7)**	**[6]**	**[6]**
ECs1561	Sp6	Absent	EspN	**-**	**-**	**-**	**-**	**[8]**	**(7)**	**(5)**	**[6]**
ECs1567	Sp6	Absent	EspO1-1	**-**	**-**	**-**	**-**	**[8]**	0	**[6]**	1
ECs1568	Sp6	Absent	EspR1	**-**	**-**	**-**	**-**	**[8]**	**[8]**	**[6]**	**[6]**
ECs1810	Sp9	Absent	NleG2-1	**-**	**-**	**-**	**-**	**(7)**	**(7)**	**[6]**	1
ECs1811	Sp9	Absent	NleG2-1	**-**	**-**	**-**	**-**	**(7)**	**(7)**	0	2
ECs1812	Sp9	Absent	NleA/EspI	**-**	**-**	**-**	**-**	**(7)**	**(7)**	0	1
ECs1814	Sp9	Absent	NleH1-2	**-**	**-**	**-**	**-**	4	6	**[6]**	**[6]**
ECs1815	Sp9	Absent	NleF	**-**	**-**	**-**	**-**	4	6	**[6]**	**[6]**
ECs1824	Sp9	Absent	NleG	**-**	**-**	**-**	**-**	**[8]**	**(7)**	**[6]**	0
ECs1825	Sp9	Absent	EspM1	**-**	**-**	**-**	**-**	**[8]**	**(7)**	**[6]**	0
ECs2226	Sp12	Absent	NleG7	**-**	**-**	**-**	**-**	**[8]**	**[8]**	**[6]**	**[6]**
ECs2714	Sp14	Absent	EspJ	**-**	**-**	**-**	**-**	**[8]**	0	0	1
ECs3485	Sp17	Absent	EspM2	**-**	**-**	**-**	**-**	**[8]**	**(7)**	**[6]**	2
ECs3486	Sp17	Absent	NleG8-2	**-**	**-**	**-**	**-**	**[8]**	**(7)**	**[6]**	3
ECs3487	Sp17	Absent	EspW	**-**	**-**	**-**	**-**	**[8]**	**(7)**	**[6]**	3
ECs3855	SpLE3	Absent	EspL2	**-**	**-**	**-**	**-**	**[8]**	**[8]**	**[6]**	**[6]**
ECs3857	SpLE3	Absent	NleB1	**-**	**-**	**-**	**-**	**[8]**	**[8]**	**[6]**	**[6]**
ECs3858	SpLE3	Absent	NleE	**-**	**-**	**-**	**-**	**[8]**	**[8]**	**[6]**	**[6]**
ECs4653	S-loop252	Absent	EspY4	**-**	**-**	**-**	**-**	**[8]**	0	0	0
RF004	Sp4, 14	Absent	Tccp, TccP2	**-**	**-**	**-**	**-**	**[8]**	1	2	4
RF067	Sp10, 11	Absent	NleG2-2, NleG2-3	**-**	**-**	**-**	**-**	**[8]**	**[8]**	**[6]**	4
RF069	Sp10, 11, 17	Absent	NleG6-1, NleG6-2, NleG6-3	**-**	**-**	**-**	**-**	**[8]**	**(7)**	**[6]**	3
RF070	Sp10, 11	Absent	NleG5-1, NleG5-2	**-**	**-**	**-**	**-**	**[8]**	**(7)**	**(5)**	1
											
**Plasmid encoded**											
pO157_01	pO157	Absent	Metalloprotease StcE	**-**	**-**	**-**	**-**	**[8]**	0	0	0
pO157_02	pO157	Absent	Type II secretion pathway related protein	**-**	**-**	**-**	**-**	**[8]**	1	0	3
pO157_03	pO157	Absent	Type II secretion pathway related protein	**-**	**-**	**-**	**-**	**[8]**	1	0	3
pO157_04	pO157	Absent	Type II secretion pathway related protein	**-**	**-**	**-**	**-**	**[8]**	1	0	3
pO157_05	pO157	Absent	Type II secretion pathway related protein	**-**	**-**	**-**	**-**	**[8]**	0	0	2
pO157_06	pO157	Absent	Type II secretion pathway related protein	**-**	**-**	**-**	**-**	**[8]**	2	0	3
pO157_07	pO157	Absent	Type II secretion pathway related protein	**-**	**-**	**-**	**-**	**[8]**	1	0	3
pO157_08	pO157	Absent	Type II secretion pathway related protein	**-**	**-**	**-**	**-**	**[8]**	0	0	3
pO157_09	pO157	Absent	Type II secretion pathway related protein	**-**	**-**	**-**	**-**	**[8]**	1	0	3
pO157_10	pO157	Absent	Type II secretion pathway related protein	**-**	**-**	**-**	**-**	**[8]**	0	0	1
pO157_11	pO157	Absent	Type II secretion pathway related protein	**-**	**-**	**-**	**-**	**[8]**	1	0	3
pO157_12	pO157	Absent	Type II secretion pathway related protein	**-**	**-**	**-**	**-**	**[8]**	1	0	3
pO157_13	pO157	Absent	Type II secretion pathway related protein	**-**	**-**	**-**	**-**	**[8]**	0	0	1
pO157_14	pO157	Absent	Type II secretion pathway related protein	**-**	**-**	**+**	**+**	**[8]**	0	0	1
pO157_17	pO157	Absent	Hemolysin C	**-**	**-**	**-**	**-**	**[8]**	**[8]**	**(5)**	3
pO157_18	pO157	Absent	Hemolysin A	**-**	**-**	**-**	**-**	**[8]**	**(7)**	**(5)**	4
pO157_19	pO157	Absent	Hemolysin B	**-**	**-**	**-**	**-**	**[8]**	**(7)**	**(5)**	4
pO157_20	pO157	Absent	Hemolysin D	**-**	**-**	**-**	**-**	**[8]**	**(7)**	**(5)**	4
pO157_39	pO157	Absent	Hemagglutinin-associated protein	**-**	**+**	**-**	**-**	**[8]**	5	0	1
pO157_59	pO157	Absent	Putative adherence factor, Efa1 homolog	**-**	**-**	**-**	**-**	**[8]**	3	0	**(5)**
pO157_76	pO157	Absent	KatP	**-**	**-**	**-**	**-**	6	6	**(5)**	2
pO157_79	pO157	Absent	EspP	**-**	**-**	**-**	**-**	**[8]**	**(7)**	**[6]**	4
pO157_80	pO157	Absent	Putative polysaccharide deacetylase (ecf1)	**-**	**-**	**-**	**-**	**[8]**	**(7)**	**(5)**	4
pO157_81	pO157	Absent	Putative LPS-1,7-N-acetylglucosamine transferase (ecf2)	**-**	**-**	**-**	**-**	**[8]**	**(7)**	**(5)**	4
pO157_82	pO157	Absent	Putative membrane protein (ecf3)	**-**	**-**	**-**	**-**	**[8]**	**(7)**	**(5)**	4
pO157_83	pO157	Absent	Putative lipid A myristoyl transferase, MsbB2 (ecf4)	**-**	**-**	**-**	**-**	**[8]**	**(7)**	**(5)**	4

*Conservation of O157 Sakai virulence genes in four sequenced pathogenic *E. coli *strains was determined according to the results of homology search using the BLASTP program. The threshold for presence (+) or absence (-) determination was ≥90% sequence identity and ≥50% aligned length coverage of a query sequence. ^† ^Genes conserved in all strains are indicated by brackets in bold, and those absent only in one strain by parentheses in bold.

Iron uptake systems are also important for bacterial survival in host environments. O157 Sakai contains seven gene clusters for iron uptake. All were conserved in every O157 strain except for strains 4 and 7, where locus 4 was missing (Table 5). In non-O157 EHECs, although three clusters common with K-12 were present in all strains, another four clusters were completely missing.

**Table 5:** Conservation of the loci for iron uptake systems in each EHEC strain

		K-12	O157								O26								O111						O103					
						
Locus no.	ECs number		#2	#3	#4	#5	#6	#7	#8	#9	#1	#2	#3	#4	#5	#6	#7	#8	#1	#2	#3	#4	#5	#6	#1	#2	#3	#4	#5	#6
1	ECs0154-0157, 1752, 3889, 3890	**+**	**+**	**+**	**+**	**+**	**+**	**+**	**+**	**+**	**+**	**+**	**+**	**+**	**+**	**+**	**+**	**+**	**+**	**+**	**+**	**+**	**+**	**+**	**+**	**+**	**+**	**+**	**+**	**+**
2	ECs0413-0415	**-**	**+**	**+**	**+**	**+**	**+**	**+**	**+**	**+**	**-**	**-**	**-**	**-**	**-**	**-**	**-**	**-**	**-**	**-**	**-**	**-**	**-**	**-**	**-**	**-**	**-**	**-**	**-**	**-**
3	ECs0622-0635	**+**	**+**	**+**	**+**	**+**	**+**	**+**	**+**	**+**	**+**	**+**	**+**	**+**	**+**	**+**	**+**	**+**	**+**	**+**	**+**	**+**	**+**	**+**	**+**	**+**	**+**	**+**	**+**	**+**
4	ECs1693-1699	**-**	**+**	**+**	**-**	**+**	**+**	**-**	**+**	**+**	**-**	**-**	**-**	**-**	**-**	**-**	**-**	**-**	**-**	**-**	**-**	**-**	**-**	**-**	**-**	**-**	**-**	**-**	**-**	**-**
5	ECs3913-3917	**-**	**+**	**+**	**+**	**+**	**+**	**+**	**+**	**+**	**-**	**-**	**-**	**-**	**-**	**-**	**-**	**-**	**-**	**-**	**-**	**-**	**-**	**-**	**-**	**-**	**-**	**-**	**-**	**-**
6	ECs4250&4251	**+**	**+**	**+**	**+**	**+**	**+**	**+**	**+**	**+**	**+**	**+**	**+**	**+**	**+**	**+**	**+**	**+**	**+**	**+**	**+**	**+**	**+**	**+**	**+**	**+**	**+**	**+**	**+**	**+**
7	ECs4380-4387	**-**	**+**	**+**	**+**	**+**	**+**	**+**	**+**	**+**	**-**	**-**	**-**	**-**	**-**	**-**	**-**	**-**	**-**	**-**	**-**	**-**	**-**	**-**	**-**	**-**	**-**	**-**	**-**	**-**

Symbols: '+' indicates a locus where all genes were conserved; '-' a locus where all genes were absent. Genes judged as 'uncertain' were not considered.

LEE is a T3SS-encoding pathogenicity island (SpLE4 in O157 Sakai) acquired by lateral gene transfer (LGT). Although LEE has been found in various EHEC and EPEC strains, they are genetically divergent. Based on the sequence polymorphism of the *eaeA *gene encoding intimin, 28 alleles have been identified so far [34]. Although the core regions of each type of LEE encode nearly the same set of genes, their DNA sequences are known to be significantly divergent. For example, the sequence identity of the LEE core region between O157 Sakai (intimin γ1) and the O26:NM strain 413/89-1 (intimin β1) (accession number: AJ277443) is around 93% on average, and that between O157 Sakai and the O103:H2 strain RW1374 (intimin ε) [35] (accession number: AJ303141) is also 93%. In our CGH analysis, many probes for LEE core genes exhibited reduced signal intensities, just below borderline for presence/absence calls in all the non-O157 EHEC strains, and thus many LEE core genes were judged as 'absent' (Table 4). This indicates that the core genes of the non-O157 EHEC strains, which include seven LEE-encoded effector genes, also have significantly diverged nucleotide sequences.

Of the 32 non-LEE effectors, all but three are encoded on Sps and SpLEs [15]. These non-LEE effectors on Sps and SpLEs, which are composed of 22 singleton genes and 4 repeated gene families, exhibited an unexpectedly high level of conservation in non-O157 EHECs. Six were conserved in all strains, eighteen in more than half of the strains, and all in at least one strain (Table 4). In contrast, three non-LEE effectors on non-prophage regions were fully absent in all non-O157 EHEC strains.

### Plasmid-encoded virulence-related genes

O157 Sakai contains a 93 kb virulence plasmid (pO157) and a small cryptic plasmid (pOSAK1) [36]. As previously reported [18], genes on pO157 were almost completely conserved in O157 strains excepted for strain 2, where 18 genes were missing. In contrast, these plasmid genes exhibited poor and highly variable conservation patterns in the non-O157 EHEC strains (Figures 1 and 2). Consistent with the plasmid profiles, all the pO157 genes except for an IS-related gene were absent in O26 strain 1 and O103 strain 2, in which no large plasmid was detected (Table 2; Additional data file 3). In other non-O157 EHEC strains that contained one or more large plasmids, pO157 genes were variably conserved: percentages of genes judged as 'present' in each strain ranged from 18% to 59%.

Importantly, genes for enterohemolysin, KatP catalase, and EspP protease, all of which are suspected to be involved in O157 virulence, were also well conserved in non-O157 EHECs (Table 4). The *ecf *operon (*ecf1 *to *ecf4*), encoding a lipid A modification system that has recently been found to be related to colonization of bovine intestine [37], was also well conserved in the non-O157 EHEC strains.

### Comparative analysis of genomic structures in EHEC strains by WGPScanning

Although the gene composition of each strain can be easily analyzed by CGH, it does not provide positional information, such as strain-specific translocations and strain-specific insertions. To obtain more details on the genomic differences between O157 and non-O157 EHECs, we analyzed the non-O157 EHEC strains by WGPScanning, and compared the results with earlier information on O157 strains [19] (Figures 1 and 2). Remarkable structural variations had been found mainly in Sp and SpLE regions in the O157 strains. In the non-O157 EHEC strains, Sp and SpLE regions exhibited much higher levels of structural change, and various other chromosomal loci containing S-loops also showed remarkable structural alterations. Because the PCR products obtained from most of these loci were reduced in size, we consider that S-loops have been deleted. This supposition is in good agreement with the CGH data.

We were able to obtain PCR products rarely from most Sp and SpLE regions in the non-O157 EHEC strains. Only SpLE1 and Sp10 regions of a few non-O157 EHEC strains yielded PCR products from their entire regions, indicating that only these strains contained genetic elements closely related to SpLE1 and Sp10 at the same loci as in Sakai. At other Sp- and SpLE-integration sites, it is likely that no insertion exists or different types of genomic elements have been inserted. We performed further PCR analyses to confirm this, using primer pairs targeting the flanking regions of each Sp and SpLE. We obtained PCR products from many Sp and SpLE loci through this analysis, and the results suggest that no large insertions exist at these loci (indicated by blank areas in Figures 1 and 2). At the remaining sites, it appears that large inserts different from those of O157 Sakai have been integrated. Of interest was the finding that no PCR product was obtained for many genes detected by the CGH analysis on the Sp and SpLE loci (see the Sp4 region of Figure 1 as an example). These results indicate that non-O157 EHEC strains also contain Sp- and SpLE-like elements, which are structurally and/or positionally highly divergent from those in O157 Sakai.

In non-prophage regions, a number of segments (49 in total) were again not amplified by PCR, suggesting that these loci contain large insertions or some other types of genomic rearrangements (indicated by arrowheads in Figures 1 and 2). In these regions, we identified several alternative integration sites for LEEs and Stx phages, as described in the next section.

Although a significant number of pO157 genes were detected in the CGH analysis, pO157-targeted primer pairs yielded no PCR product in all the non-O157 EHEC strains with a single exception (a small segment in one O26 strain; Figures 1 and 2). This indicates that plasmids harbored by non-O157 EHEC strains are highly divergent from pO157 in structure.

### Integration sites of Stx phages and LEE islands

**Figure 5:**
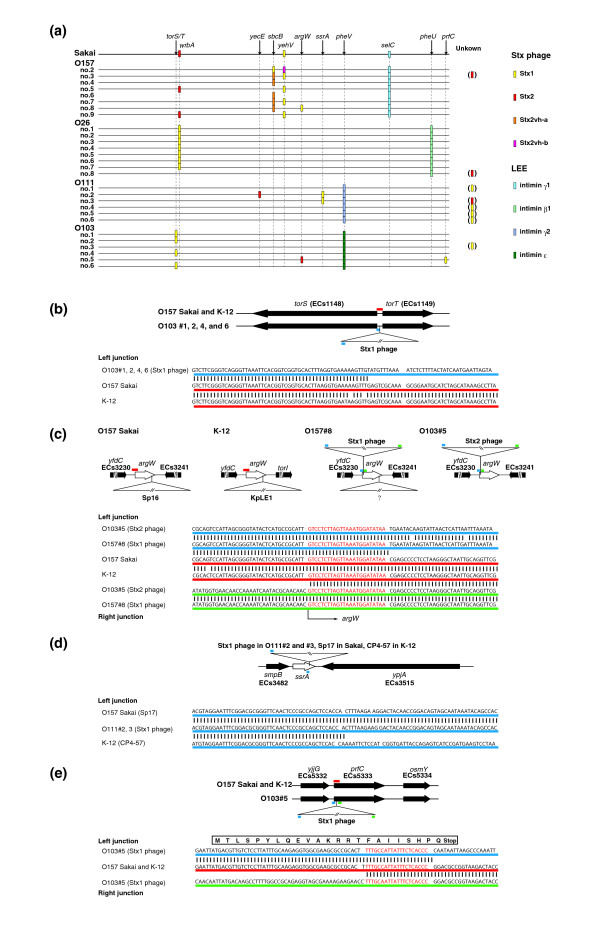
Variation in the integration sites for Stx phages and LEE islands in O157 and non-O157 EHEC strains. **(a) **Locations of the Stx phages and the LEE islands on each chromosome are shown. Integration sites of Stx2 phages in O157 strain 3, O26 strain 8, and O111 strain 3, and those of Stx1 phages in O111 strains 1, 4, 5, and 6, and O103 strain 3 are unknown. **(b-e) **Schematic presentation of newly identified integration sites for Stx phages. DNA sequences of left or right junctions for each integration site are also shown. (b) The *torS*-*tort *intergenic region in O103 strains 1, 2, 4, and 6. The *torS *and *torT *genes encode a sensor for a two-component regulatory system and a periplasmic protein of unknown function, respectively. The right junction was not identified. (c) The *argW *region in O157 strain 8 and O103 strain 5. The *argW *gene encodes an arginine tRNA. (d) The *ssrA *region in O111 strains 2 and 3. The *ssrA *gene encodes the tmRNA. The right junction was not identified. (e) The *prfC *gene in O103 strain 5. The *prfC *gene encodes the peptide chain release factor (RF-3). Integration of Stx1 phage into the *prfC *gene changes the amino acid sequence of a short amino-terminal region of RF-3, and removes the authentic ribosome binding site and promoter sequences. It is not known whether the *prfC *gene is transcribed and/or translated in the strain. The *prfC *gene, however, is not listed as an essential gene of *E. coli *[24].

All the non-O157 EHEC strains examined in this study carried Stx phage(s) and the LEE. The results of WGPScanning analyses, however, implied that their integration sites are different from those in O157 Sakai (Figures 1 and 2). We thus searched for integration sites of these elements in the non-O157 EHEC strains. We first searched for LEE integration sites by long PCR using primer pairs, one targeting *eaeA *and the other the flanking regions of known LEE integration sites. This analysis revealed that LEEs are located at the *pheU *locus in all O26 strains and the *pheV *locus in all O111 and O103 strains (Figure 5a).

Although Stx1 and Stx2 phages are integrated into the *wrbA *and *yehV *genes, respectively, in the two sequenced O157 strains (Sakai and EDL933), several alternative integration sites of Stx phages have been described in other O157 strains; one site for Stx1 phage (*sbcB*) and two for Stx2 phages (*sbcB *and *yehV*) [19]. The *yecE *locus has also been identified as an integration site of the Stx2 phage in an ONT:H - strain [38]. We consecutively analyzed these sites of the non-O157 EHEC strains by long PCR using primer pairs specific to *stx1A *(or *stx2A*) and each of these integration sites. We could find Stx1 phages at the *wrbA *locus in only seven O26 strains (1 to 7) and a Stx2 phage at the *yecE *locus in only one O111 strain (strain 2) (Figure 5a). We then constructed fosmid libraries of six EHEC strains (O157 strain 8, O26 strain 2, O111 strains 2 and 3, and O103 strains 1 and 5), and screened for *stx1- *or *stx2-*containing clones. By this systematic screening, we identified four new integration sites (*torS*-*torT *intergenic region, *argW*, *ssrA*, and *prfC*) for Stx phages. Based on this finding, the long PCR strategy also enabled us to find the Stx1 phages integrated at the *torS-torT *intergenic region in three O103 strains (2, 4, and 6) (Figure 5a). These results indicate that Stx phages are extremely divergent not only in genomic structure but also in integration site among EHEC strains. DNA sequences of these newly identified integration sites are shown in Figure 5b-e).

## Discussion

A previous whole genome comparison of two *E. coli *strains, K-12 and O157 Sakai, revealed that their chromosomes are large mosaics of conserved core sequences and strain-specific sequences [8]. Our present CGH analysis of O157 and non-O157 EHEC strains demonstrates that the core sequences are also well conserved in the non-O157 EHEC strains. Although the EHEC strains analyzed here were derived from three different clonal lineages, 3,240 out of 3,651 'conserved in K-12' singleton genes and 11 out of 23 'conserved in K-12' repeated gene families were perfectly conserved in all EHEC strains. The number of '*E. coli *core genes' proposed by several array-based genome comparisons ranges from 2,800 to 3,782 genes [39-43]. The difference would come from the number and types of tested strains and types of microarrays used in each study.

More than 1,600 O157 Sakai genes that are absent from K-12 encode a great variety of proteins, including various virulence factors. Most of these 'Sakai-specific' genes are also absent from four sequenced extra-intestinal pathogenic *E. coli *strains (Table 4; Additional data file 4), which belong to a different phylogenetic group (Additional data file 2). The highly biased GC content of 'Sakai-specific' genes implies that most of them have been acquired by LGT [8]. In fact, two-thirds of the S-loops are prophages and prophage-like elements. Our CGH analysis demonstrated a very poor conservation of 'Sakai-specific' genes in non-O157 EHECs: only 98 'Sakai-specific' singleton genes were conserved in all tested EHEC strains (Figure 3; Additional data file 4). Among these, 16 genes were also present in all the sequenced extra-intestinal pathogenic *E. coli *strains, indicating that they have been specifically deleted in the K-12 lineage.

Interestingly, however, a significant number of virulence-related genes, particularly those for non-LEE effectors and non-fimbrial adhesins, were well conserved in the non-O157 EHEC strains (Table 4). All four sequenced extra-intestinal pathogenic strains do not contain homologues of 31 non-LEE effectors and 11 non-fimbrial adhesins that are absent in K-12, except for three non-fimbrial adhesin genes (ECs0350, Ecs0362, and ECs1360). It is thus assumed that these virulence-related genes were selectively acquired and retained in multiple EHEC lineages like the genes for Stx, LEE, and enterohemolysin. Most of these virulence genes are on prophages, prophage-like elements, or the plasmid [8,15,44]. 'Sakai-specific' repeated gene families derived from prophages were also well conserved in the non-O157 EHEC strains, suggesting that they contain multiple prophages similar to those of O157 Sakai. These results indicate that infection of similar bacteriophages is deeply involved in the evolution of O157 and non-O157 EHEC lineages.

Prophages in non-O157 EHEC strains are remarkably divergent in their structure and integration site from those in O157 Sakai. With respect to this, another important achievement of this study is the identification of a set of alternative integration sites for prophages and other large genomic elements in the non-O157 EHEC genomes (Figures 1 and 2). They include those for the Stx phages and LEE islands (Figure 5).

Selective conservation of pO157-associated virulence-related genes in non-O157 EHECs is also intriguing. Most non-O157 EHEC strains contained one or two large plasmids, but their structures were very different from pO157, and pO157 genes other than virulence-related genes were very poorly conserved in non-O157 EHEC strains. (Figures 1 and 2; Table 4). Although more information on the large plasmids of non-O157 EHECs is necessary, similar but significantly diverged plasmids may have independently carried these virulence-determinants into each EHEC lineage.

The genome sizes of the non-O157 EHEC strains were similar or rather larger than those of O157 strains (Table 2). The CGH data, on the other hand, indicates that 83% of 4,905 singleton genes and 78% of 151 repeated gene families (composed of 542 repeated genes) are conserved in non-O157 EHEC strains on average. Taken together, we can expect that each non-O157 EHEC strain contains around 950 serotype- or strain-specific genes that do not exist in O157 Sakai. The presence of such a huge amount of serotype- or strain-specific genes may explain why non-O157 EHECs exhibit several phenotypes distinct from O157. For example, O26, O111, and O103 EHEC can cause diseases in cattle, goats, pigs, and rabbits, while O157 rarely does [6]. In this regard, the absence of several gene clusters for fimbrial biosynthesis and iron utilization systems in non-O157 EHECs may suggest that non-O157 EHEC lineages have acquired alternative gene clusters for virulence towards these animals, which may confer different types of host tropisms to these lineages. To address these issues, more detailed analyses of non-O157 EHEC strains, including whole genome sequence determination, will be required.

## Conclusion

We describe the first systematic whole genome comparison between O157 and non-O157 EHEC strains based on the O157 Sakai sequence. Chromosomal backbone regions were highly conserved both in O157 and non-O157 EHEC strains of O26, O111, and O103 serotypes. In contrast, O157 Sakai-specific regions were very poorly conserved in the non-O157 EHEC strains, even though their total genome sizes were the same or rather larger than that of O157. It is assumed, therefore, that O157 and non-O157 EHEC strains have independently acquired a huge amount of lineage- or strain-specific genes by LGT. On the other hand, an unexpectedly large number of virulence genes, especially those for non-LEE effectors and non-fimbrial adhesions, were well conserved in non-O157 EHEC strains in addition to the *stx *genes and LEE island. In O157, most of them were encoded on prophages and the plasmid. Although non-O157 EHEC strains contained multiple prophages similar to those of O157, these prophages exhibited remarkable structural and positional diversity. These data suggest that infections of similar but distinct bacteriophages are deeply involved in the evolution of EHEC strains belonging to different *E. coli *lineages.

## Materials and methods

### Bacterial strains, growth conditions, and DNA preparation

Bacterial strains used in this study are listed in Table 1. O157 Sakai and K-12 MG1655 were used as references in CGH and WGPScanning analyses. Eight EHEC O157:H7 (or H-) strains were previously described [19]. Of the tested non-O157 EHEC strains, ED71, ED80, and ED411 were isolated in Italy (kindly provided by S Morabito, Istituto Superiore di Sanità, Rome), PMK5 in France [45], and the others in Japan in 2001. Growth conditions and the protocol for genomic DNA preparation were described previously [18].

### Detection and subtyping of *stx *and *eae *genes

Detection of *stx1 *genes was done by PCR amplification with primers stx1-F (5'-caggggataatttgtttgcagttg-3') and stx1-R (5'-gacacatagaaggaaactcatcag-3'), using 10 ng of genomic DNA as template with a EX taq PCR kit (Takara Bio, Kyoto, Japan) by 30 amplification cycles of denaturation for 20 s at 98°C, annealing for 30 s at 60°C, and primer extension for 45 s at 72°C. The amplified DNA was analyzed by electrophoresis on 2% agarose gel. Detection and subtyping of *stx2 *and *eae *were done by restriction fragment length polymorphism (RFLP) analysis of PCR products as described previously [46,47].

### Multi-locus sequence typing

Internal regions of each of seven housekeeping genes, *aspC*, *clpX*, *fadD*, *icdA*, *lysP*, *mdh*, and *uidA*, were amplified and sequenced for each test strain. Primer designs and PCR conditions were determined according to the 'multil-locus sequence typing database for pathogenic *E. coli *[48]. The sequences of seven loci were concatenated and aligned with those of other pathogenic *E. coli *strains in the EcMLST database by using the ClustalW program [49] in the MEGA3 software [50], and then a neighbor-joining (NJ) tree was generated by using the Tamura-Nei evolutionary model.

### Pulsed-field gel electrophoresis

PFGE analyses were performed according to the method described by Terajima *et al*. [51] with some modification. In brief, bacterial cells were embedded in 0.9% Certified Low Melt Agarose (Bio-Rad Laboratories, Inc., Tokyo, Japan), lysed with a buffer containing 0.2% sodium deoxycholate, 0.5% N-lauroylsarcosine, and 0.5% Brij-58, and treated with 100 μg/ml proteinase K. *Xba*I-digested genomic DNA was separated by using CHEF MAPPER (Bio-Rad Laboratories, Inc.) with 1% Pulsed Field Certified Agarose (Bio-Rad Laboratories, Inc.,) at 6.0 V/cm for 22 h and 18 minutes with pulsed times ranging from 47 to 44.69 s. I-*Ceu*I-digested DNA was with 1% Pulsed Field Certified Agarose at 6.0 V/cm for 23 h and 52 minutes with pulsed times ranging from 1.19 to 83.55 s or with 0.8% Agarose at 3.0 V/cm for 24 h with pulsed times ranging from 600 to 800 s. Sizes of each DNA band were estimated by Lane Analyzer (ATTO Corp., Tokyo, Japan).

### Plasmid profile

Plasmid DNA was purified from overnight culture of each EHEC strain using a plasmid midi kit (Qiagen, Tokyo, Japan) according to the manufacturer's instructions, and was separated by the CHEF MAPPER with 1% Pulsed Field Certified Agarose at 6.0 V/cm for 12 h with pulsed times ranging from 0.14 to 21.79 s. Band sizes were estimated by Lane Analyzer.

### Microarray analysis

The protocol of CGH analysis using an O157 oligoDNA microarray has been described previously [18]. In brief, oligonucleotide probes were prepared for all the protein-coding genes in the Sakai genome (5,447 genes in total). The probes were principally 60-mer in length and two probes were prepared for each gene. Repeated genes sharing various lengths of almost identical sequences (542 genes in total) were grouped into 151 repeated gene families, and each family was represented by a single probe. Genomic DNA (3 μg) from the reference strain (O157 Sakai) and each test strain was used to generate Cy3- and Cy5-labeled samples, respectively, and cohybridized on a single array. For each test strain, DNA labeling and hybridization were performed twice independently. Fluorescence intensities of the spots were collected using the ArrayVision 8.0 software (Imaging Research Inc., Ontario, Canada). After filtrating the spots with slide abnormalities or low signal intensities in the reference channels, the fluorescence intensity in each cannel was log_2_-transformed. Presence or absence of each probe was then determined by using the array-based genotyping software GACK [52]. The presence or absence of each gene was finally determined according to each probe result obtained from two independent hybridizations as described previously [18]. Processed datasets were displayed in genomic order using the TREEVIEW program [53].

In our previous test experiment using K-12 strain MG1655, 96.9% of the genes that were predicted as 'present' by an *in silico *analysis (threshold value ≥90% identity in each 60-mer probe sequence) were judged as 'present' in the microarray analysis, and 97.8% of the genes predicted as 'absent' were judged as 'absent' [18].

The microarray data have been submitted to the Gene Expression Omnibus (series record number GSE7931). Final processed data are presented in Additional data file 6.

### WGPScanning analysis

The WGPScanning method was described previously [19]. In brief, we used a total of 1,120 primers that can amplify the entire O157 Sakai genome by 560 long PCRs (549 for the chromosome and 11 for the plasmids). All the primer sequences are available at our web site [54]. Long PCR was performed using the LA taq PCR kit (Takara Shuzo, Kyoto, Japan) and 1 ng of genomic DNA as template with 30 cycles of a two-step amplification program: 20 s at 98°C and 16 minutes at 69°C. PCR products were separated by field inversion gel electrophoresis (FIGE), and product sizes were estimated by Lane Analyzer.

### Construction of fosmid libraries

To identify alternative integration sites of Stx phages, we constructed fosmid libraries for O157 strain 8, O26 strain 2, O111 strains 2 and 3, and O103 strains 1 and 5 by using a Copycontrol Fosmid Library Production Kit (Epicentre, Medison, WI, USA) according to the manufacturer's instructions. Insert sizes and redundancies of each library were, on average, 40 kb and 20 times, respectively. The *stx1*- and *stx2*-containing clones were screened by PCR using the following primer pairs; stx1-F, 5'-gacacatagaaggaaactcatcag-3', and stx1-R, 5'-caggggataatttgtttgcagttg-3' for *stx1*; and stx2-F, 5'-ggcgcgttttgaccatcttcgt-3', and stx2-R, 5'-tacctttagcacaatccgccgc-3' for *stx2*. End sequences of each insert were determined by direct sequencing, and were used to roughly map the integration sites on the *E. coli *chromosome. Precise integration sites were determined by the primer-walking method.

## Additional data files

The following additional data files are available with the online version of this paper. Additional file 1 is a figure showing the gel of a PFGE analysis of *Xba*I-digested genomic DNA in O157 and non-O157 EHEC strains. Additional file 2 shows the phylogeny of O157 and non-O157 EHEC strains determined by MLST. Additional file 3 is a figure showing the gel of a PFGE analysis of plasmids isolated from O157 and non-O157 EHEC strains. Additional file 4 is a summary of the CGH analyses. Additional file 5 presents the data on conservation of the 'conserved in K-12' singleton genes belonging to each COG category in the EHEC strains. Additional file 6 is a table listing all the result of CGH analyses in non-O157 EHEC strains (processed data only).

## Supplementary Material

Additional data file 1 *Xba*I-digestion patterns of EHEC genomic DNA are shown. Click here for file

Additional data file 2 The MLST analysis of EHEC strains of the present study with other pathogenic *E. coli *strains in the EcMLST database was conducted by using concatenated DNA sequences of seven loci (*aspC*, *clpX*, *fadD*, *icdA*, *lysP*, *mdh *and *uidA*). The sequences of reference strains were obtained from EcMLST. Multiple sequence alignments were made by using the ClustalW program in the MEGA3 software. The NJ tree was generated by using the Tamura-Nei evolutionary model. Bootstrap values greater than 50% are indicated. The scale bar represents the number of substitutions per site. Accession numbers in EcMLST, strain names, serotypes, and classes in EcMLST of each strain are indicated. MLST20 is an undefined clonal group. Click here for file

Additional data file 3 Plasmid profiles of O157 and non-O157 EHEC strains are shown. Click here for file

Additional data file 4 The results of CGH analyses of O157 and non-O157 EHEC strains are summarized. Click here for file

Additional data file 5 Conservation of the 'conserved in K-12' singleton genes belonging to each COG category was analyzed in each EHEC serotype. Click here for file

Additional data file 6 The processed data of CGH analyses of non-O157 EHEC strains are shown. Click here for file
