# Supplementary figures and images for: Extensive genomic diversity and selective conservation of virulence-determinants in enterohemorrhagic Escherichia coli strains of O157 and non-O157 serotypes
Source: Genome Biol. 2007 Jul 10;8(7):R138. doi: 10.1186/gb-2007-8-7-r138 (PMC2323221; doi:10.1186/gb-2007-8-7-r138)

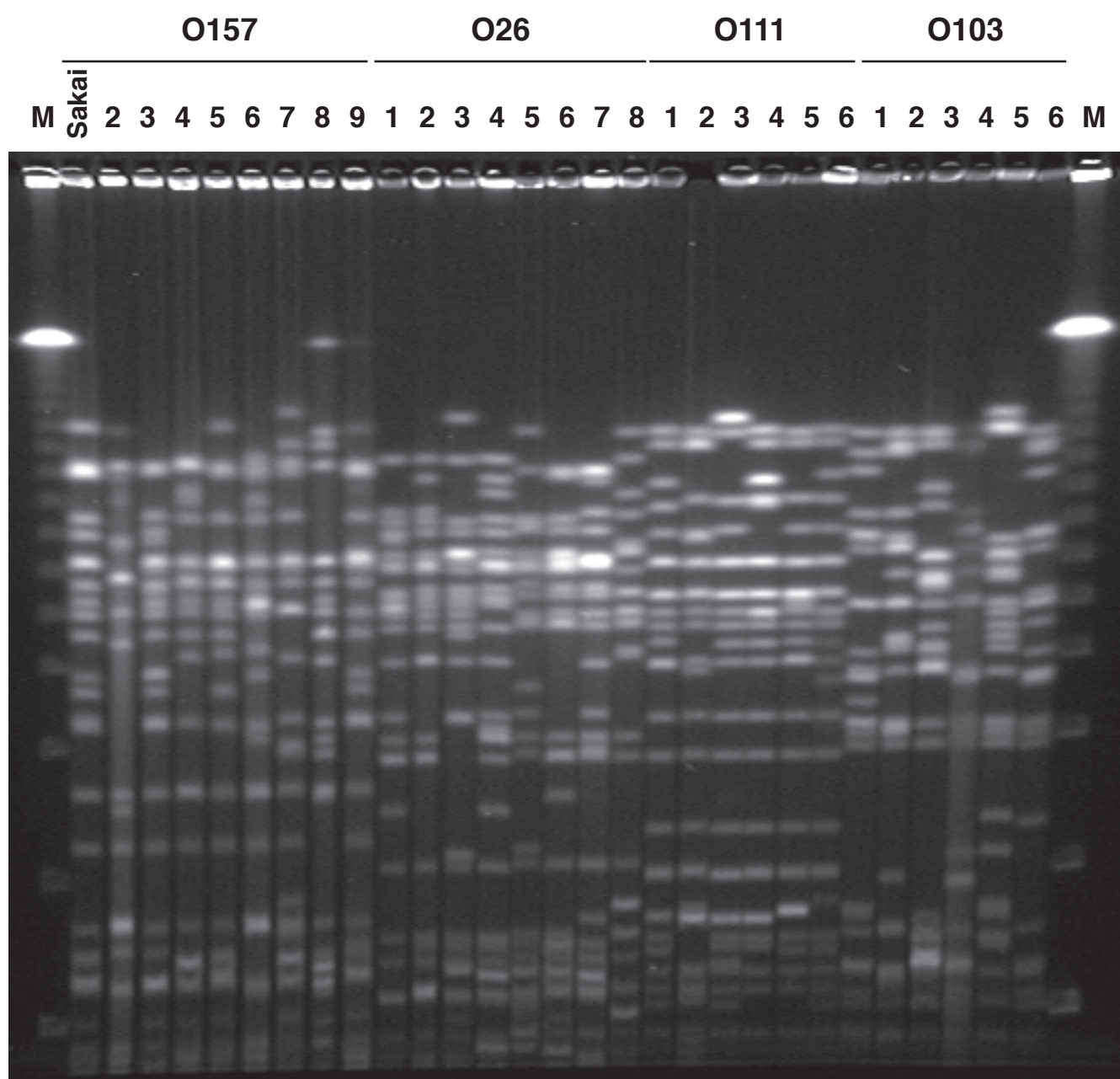

Figure S1

Supplement: Additional data file 1 — XbaI-digestion patterns of EHEC genomic DNA are shown. [file gb-2007-8-7-r138-S1.pdf]

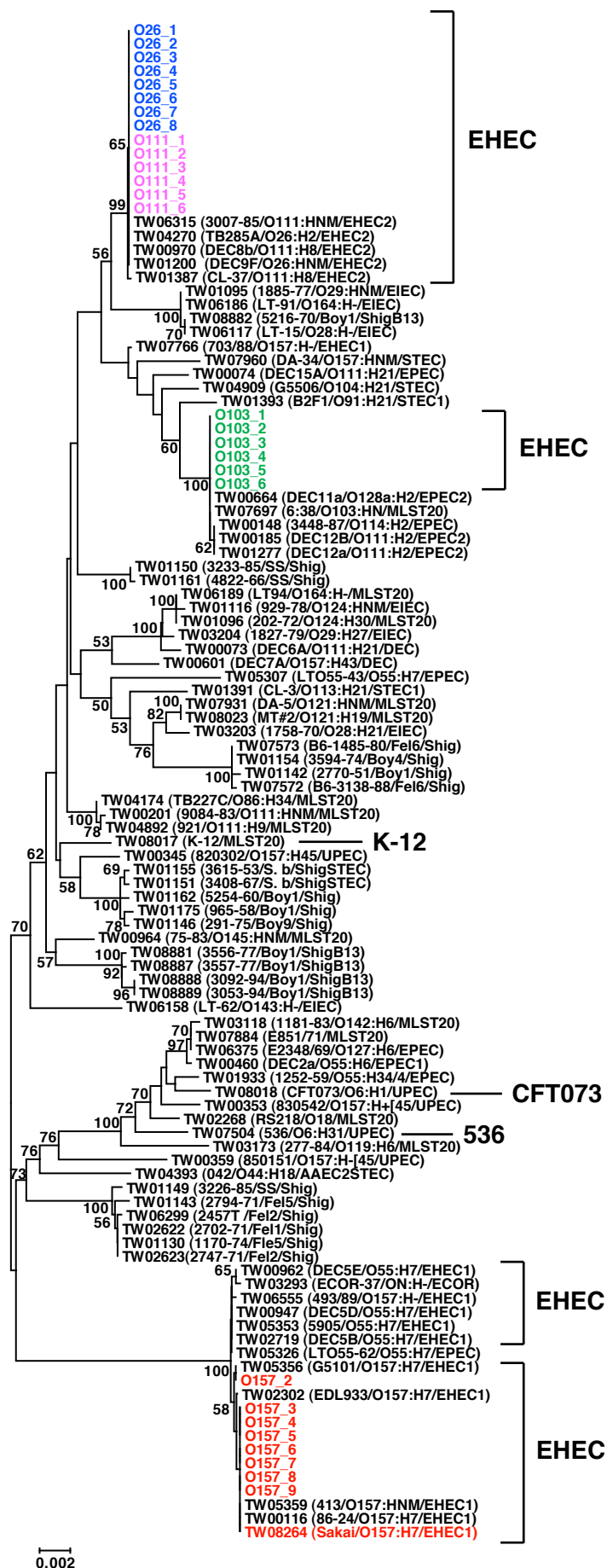

Figure S2

Supplement: Additional data file 2 — The MLST analysis of EHEC strains of the present study with other pathogenic E. coli strains in the EcMLST database was conducted by using concatenated DNA sequences of seven loci (aspC, clpX, fadD, icdA, lysP, mdh and uidA). The sequences of reference strains were obtained from EcMLST. Multiple sequence alignments were made by using the ClustalW program in the MEGA3 software. The NJ tree was generated by using the Tamura-Nei evolutionary model. Bootstrap values greater than 50% are indicated. The scale bar represents the number of substitutions per site. Accession numbers in EcMLST, strain names, serotypes, and classes in EcMLST of each strain are indicated. MLST20 is an undefined clonal group. [file gb-2007-8-7-r138-S2.pdf]

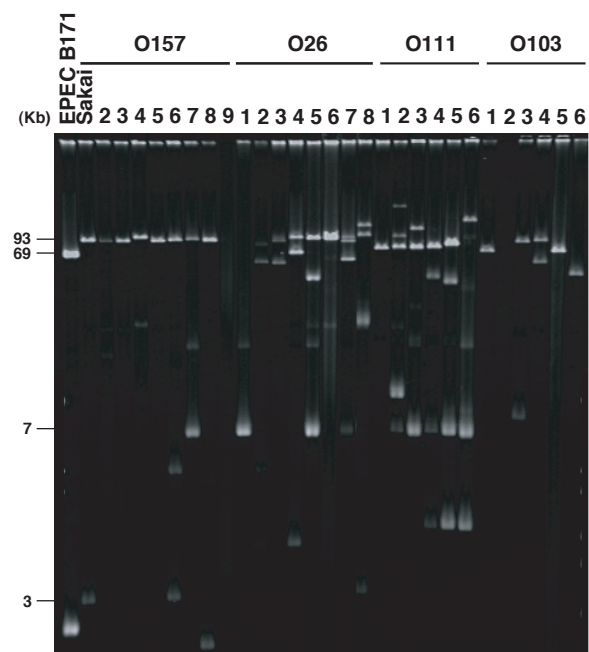

Figure S3

Supplement: Additional data file 3 — Plasmid profiles of O157 and non-O157 EHEC strains are shown. [file gb-2007-8-7-r138-S3.pdf]
