# Supplementary material for: Extensive genomic diversity and selective conservation of virulence-determinants in enterohemorrhagic Escherichia coli strains of O157 and non-O157 serotypes
Source: Genome Biol. 2007 Jul 10;8(7):R138. doi: 10.1186/gb-2007-8-7-r138 (PMC2323221; doi:10.1186/gb-2007-8-7-r138)
Supplement: Additional data file 4 — The results of CGH analyses of O157 and non-O157 EHEC strains are summarized. [file gb-2007-8-7-r138-S4.pdf]

Table S1: Summary of microarray analyses of EHEC strains.

	Number of genes (%)																				
	in silico*								**CGH data****												
	**CFT073**		**UTI89**		**536**		**APEC**		**O157**			**O26**			**O111**			**O103**			**FC in all EHEC**
	**Present**	**Absent**	**Present**	**Absent**	**Present**	**Absent**	**Present**	**Absent**	**FC**	**VAP**	**FA**	**FC**	**VAP**	**FA**	**FC**	**VAP**	**FA**	**FC**	**VAP**	**FA**	
**Singleton genes**																					
"conserved in K-12" genes [3,651 genes]	3,261 **(89.3)**	390 **(10.7)**	3,300 **(90.4)**	351 **(9.6)**	3,261 **(89.3)**	390 **(10.7)**	3,117 **(85.4)**	534 **(14.6)**	3,596 **(98.5)**	55 **(1.5)**	0	3,450 **(94.5)**	155 **(4.2)**	46 **(1.3)**	3,331 **(91.2)**	237 **(6.5)**	83 **(2.3)**	3,542 **(97.0)**	79 **(2.2)**	30 **(0.8)**	3,240 **(88.7)**
"partly conserved in K-12" genes [101 genes]	69 **(68.3)**	32 **(31.7)**	71 **(70.3)**	30 **(29.7)**	70 **(69.3)**	31 **(30.7)**	68 **(67.3)**	33 **(32.7)**	96 **(95.0)**	5 **(5.0)**	0	44 **(43.6)**	48 **(47.5)**	9 **(8.9)**	40 **(39.6)**	50 **(49.5)**	11 **(10.9)**	58 **(57.4)**	39 **(38.6)**	4 **(4.0)**	37 **(36.6)**
"Sakai-specific" genes [1,153 genes]	162 **(14.1)**	991 **(85.9)**	204 **(17.7)**	949 **(82.3)**	122 **(10.6)**	1,031 **(89.4)**	158 **(13.7)**	995 **(86.3)**	741 **(64.3)**	412 **(35.7)**	0	221 **(19.2)**	458 **(39.7)**	474 **(41.1)**	300 **(26.0)**	275 **(23.9)**	578 **(50.1)**	231 **(20.0)**	535 **(46.4)**	387 **(33.6)**	98 **(8.5)**
Total [4,905]	3,492 **(71.2)**	1,413 **(28.8)**	3,575 **(72.9)**	1,330 **(27.1)**	3,453 **(70.4)**	1,452 **(29.6)**	3,343 **(68.2)**	1,562 **(31.8)**	4,433 **(90.4)**	472 **(9.6)**	0	3,715 **(75.7)**	661 **(13.5)**	529 **(10.8)**	3,671 **(74.8)**	562 **(11.5)**	672 **(13.7)**	3,831 **(78.1)**	668 **(13.6)**	421 **(8.6)**	3,375 **(68.8)**
**Repeated gene families*****																					
"conserved in K-12" gene families [23 families]	14 **(60.9)**	9 **(39.1)**	13 **(56.5)**	10 **(43.5)**	10 **(43.5)**	13 **(56.5)**	10 **(43.5)**	13 **(56.5)**	15 **(65.2)**	8 **(34.8)**	0	15 **(65.2)**	8 **(34.8)**	0	13 **(56.5)**	10 **(43.5)**	0	17 **(73.9)**	6 **(26.1)**	0	11 **(47.8)**
"Sakai-specific" gene families [128 families]	47 **(35.7)**	81 **(64.3)**	41 **(32.0)**	87 **(68.0)**	25 **(19.3)**	103 **(80.7)**	28 **(21.9)**	100 **(78.1)**	81 **(63.3)**	47 **(36.7)**	0	74 **(57.8)**	54 **(42.2)**	0	60 **(46.9)**	68 **(53.1)**	0	77 **(60.2)**	51 **(39.8)**	0	33 **(25.8)**
Total [151 families]	61 **(40.4)**	90 **(59.6)**	54 **(35.8)**	97 **(64.2)**	35 **(22.2)**	116 **(77.8)**	38 **(25.2)**	113 **(74.8)**	96 **(63.6)**	55 **(36.4)**	0	89 **(58.9)**	63 **(41.7)**	0	73 **(48.3)**	78 **(51.7)**	0	94 **(62.3)**	57 **(37.7)**	0	44 **(29.1)**

*Conservation of O157 Sakai virulence genes in four sequenced pathogenic *E. coli* strains was determined according to the results of homology search using the BLASTP program. The threshold for presence (+) or absence (-) determination was ≥ 90% sequence identity and ≥ 50% aligned length coverage of a query sequence.

**Genes judged as 'present' in all the strain tested were categorized as 'FC (fully conserved)', thoseas 'absent' in all the strains as 'FA (fully absent)', and others as 'VAP (variably absent or present)'.

*** In the CGH analysis, repeated gene families which had reduced copy numbers are also judged as ‘absent’. Therefore, all the repeated gene families judged as 'absent' are categorized as 'uncertain'.
