# Supplementary material for: Extensive genomic diversity and selective conservation of virulence-determinants in enterohemorrhagic Escherichia coli strains of O157 and non-O157 serotypes
Source: Genome Biol. 2007 Jul 10;8(7):R138. doi: 10.1186/gb-2007-8-7-r138 (PMC2323221; doi:10.1186/gb-2007-8-7-r138)
Supplement: Additional data file 5 — Conservation of the 'conserved in K-12' singleton genes belonging to each COG category was analyzed in each EHEC serotype. [file gb-2007-8-7-r138-S5.pdf]

Table S2: "conserved in K-12" singleton genes that are absent in at least one strain in each serogroup.

COG category	No. of genes absent in at least one strain of				
	O157	O26	O111	O103	EHEC
Cell wall/membrane biogenesis [198]	2	2	16	3	18
Cell motility [76]	1	1	16	1	17
Carbohydrate transport, metabolism [314]	4	10	12	7	16
Replication, recombination, repair [147]	6	2	10	7	13
Signal transduction [129]	0	2	9	4	12
Transcription [235]	1	3	9	2	11
Amino acid transport, metabolism [328]	0	4	9	3	10
Energy production, conversion [262]	0	3	5	1	6
Inorganic ion transport, metabolism [169]	1	4	4	2	5
Nucleotide transport, metabolism [86]	0	2	1	2	3
Posttranslational modification [126]	0	0	3	0	3
Cell cycle control [34]	0	1	2	1	2
Translation [167]	2	0	1	0	2
Coenzyme transport, metabolism [118]	0	1	0	0	1
Intracellular trafficking, secretion [30]	0	1	1	0	1
Defense mechanisms [42]	1	0	0	0	1
Unkown, not in COGs [1,058]	19	39	86	28	103
ToTal [3,651]	37	75	184	61	224
